# Cover-free families on hypergraphs and combinatorial group testing

**DOI:** 10.1007/s10878-026-01429-0

**Published:** 2026-06-14

**Authors:** Thaís Bardini Idalino, Lucia Moura

**Affiliations:** 1 Universidade Federal de Santa Catarina, Florianópolis, Brazil; 2 University of Ottawa, Ottawa, Canada

**Keywords:** Combinatorial group testing, Cover-free family, Superimposed code, Disjunct matrix, Hypergraphs

## Abstract

Combinatorial group testing (CGT) is used to identify a subset of defective items from a set of items by grouping them together and performing a small number of tests on the groups. Cover-free families (CFFs), also called superimposed codes, are well-studied combinatorial structures used to design the groups in such a way that identifying the defective items from test results (decoding) can be done efficiently. This paper focuses on generalizations of CFFs that take into account a known structure among items to be tested. This structure is modeled by a hypergraph, where vertices are items to be tested and edges represent a predictable relationship among items. A typical application is testing for an infectious disease in a population where there are clusters of individuals with high contact rates, such as households within a neighbourhood or students taking courses within a school. As we aim at minimizing the number of tests, CFFs on hypergraphs yield an interesting combinatorial optimization problem and, like CFFs, have connections to coding theory, design theory and extremal set theory. In this paper, we discuss various types of CFFs on hypergraphs, decoding algorithms, bounds and constructions. In particular, we give several constructions that use the structure and properties of the underlying hypergraph.

## Introduction

In combinatorial group testing (CGT), we have *n* items to be tested, and we assume that at most *d* of them are defective. We group items into subsets and test the subsets. If the test result is negative, all items in the subset are non-defective, while a positive result indicates that there is at least one defective item in the subset. Based on the test results, we can identify up to *d* defective items. The goal is to minimize the number of tests *t* for a given *n* and *d*. Combinatorial group testing can be adaptive and non-adaptive. In adaptive CGT, the outcome of previous tests is used to decide the next tests, while in non-adaptive CGT, all tests are decided in advance (Du and Hwang 2000, 2006).

We focus on *non-adaptive* CGT, where the design of the groups can be done using *cover-free families* (CFF). A *d*-*cover-free family* is a set system where no subset is contained in the union of *d* other subsets (Erdös et al. 1985). A *d*-CFF(*t*, *n*) can be represented by a $t\times n$ binary matrix indicating the incidence of *t* points on *n* subsets of points satisfying this defining property. They are used in CGT by associating the items to be tested to columns and the groups/tests to rows, with a 1 in the matrix indicating that a test contains an item. The defining property guarantees an efficient identification of up to *d* defective items among *n* items using *t* tests in *O*(*tn*) time, as it will be detailed in Sect. 2. For a recent survey on CFFs, see Idalino and Moura (2024).

In this paper, we consider a generalization of CGT where defective items are more likely to appear together in predictable subsets of items. We consider *CFFs on hypergraphs*, where the CFF defining property is not enforced for all combinations of *d* items, but only for those associated with the edges of a given hypergraph. We define the hypergraph as follows: the vertices are the items to be tested, and the hyperedges are the predictable subsets of elements obtained directly from the application. For example, if we want to monitor a highly transmissible disease among students in a school, the students represent the vertices, and the classrooms represent the edges. When we have an infected classroom (edge), many students in that classroom are likely infected. We suggest that CFFs on hypergraphs gives a more precise model for problems like these, when compared to traditional *d*-CFFs, allowing a reduction in the number of required tests.

We define *vertex-identifying CFFs* and *edge-identifying CFFs* on hypergraphs. The first one allows the identification of all defective items, as long as they are covered by at most *r* defective edges, where *r* is a given parameter. The second one allows only the identification of the (up to *r*) defective edges, using potentially fewer tests. In this paper, we present new constructions of CFFs on hypergraphs. We consider the case of overlapping (disjoint) and non-overlapping edges and present constructions for each case. Some of them are based on traditional CFF constructions, others are based on properties of the hypergraph, such as edge and vertex colouring. We also explore what we call *Swiss-army-knife CFFs*, which are multipurpose CFFs where the same matrix can be vertex-identifying for some *r*, edge-identifying for a larger number of edges, and a traditional *d*-CFF for some *d*.

### Related work

Group testing over hypergraphs has been recently explored by Nikolopoulos et al. (2021a, 2021b, 2023) under the name of *community-aware* group testing in the context of COVID-19 testing. They explore the case of disjoint edges in (Nikolopoulos et al. 2021a) and overlapping edges in (Nikolopoulos et al. 2021b). Their definition of the hypergraph is equivalent to ours, where edges indicate directly which items belong to the same “community”. They propose adaptive and non-adaptive algorithms, as well as lower bounds on the number of tests, but there is not much emphasis on efficient CFF matrix constructions for the non-adaptive case. Our work differs from theirs in the sense that we focus on solutions using cover-free families on hypergraphs and provide a comprehensive study of them.

The work of Gonen et al. (2022), Vorobyev (2022), and De Bonis (2024) also consider group testing on hypergraphs, but they define the edges in a slightly different way. Their set $\mathcal {E}$ of hyperedges represents all the possible sets of defective items, and the proposed constructions allow the identification of any possible defective set that is an edge. Of course, there is an easy conversion between the models, but the reader needs to be aware that the hypergraphs considered are different. Moreover, their definition generalizes the one of *separable matrices*, while we generalize the one of CFFs (see Definitions 2.1 and 2.2). Gonen et al. (2022) consider both the adaptive and non-adaptive cases. For the non-adaptive case, their solution requires $O(d \log |\mathcal {E}|)$ tests, where *d* is the maximum size of an edge in $\mathcal {E}$. Vorobyev (2022) provides algorithms with improved bounds for adaptive and non-adaptive group testing. De Bonis (2024) focuses on non-adaptive, two-stage, and three-stage algorithms. They proposed an algorithm with $O(\frac{d}{p} \log |\mathcal {E}|)$ tests and with efficient decoding in *O*(*nt*) time, for a suitable parameter *p* related to the intersection of edges of the hypergraph.

In summary, our model differs from some others (De Bonis 2024; Gonen et al. 2022; Vorobyev 2022) in the definition of the underlying hypergraph: their edges represent all possible sets of defective items, while our model and the one by Nikolopoulos et al. (2021a, 2021b, 2023) directly represent predictable clusters of items given by the application, where up to *r* clusters can contain defective items. Both models can be used to solve non-adaptive group testing problems, but the edges will be different, and our model can potentially have fewer edges. Moreover, we benefit from using CFFs instead of separable matrices, since the former can have a guarantee of faster decoding (see Algorithm 1).

CFFs on hypergraphs seem to have been first introduced in the first author’s Ph.D. thesis (Idalino 2019) under the name of *variable* CFFs (VCFFs). There, it was motivated by applications in cryptography, which would allow for the location of clustered modifications in a signed document when using modification-tolerant digital signatures. Some definitions and results presented in the present paper have appeared in an early version published in a conference in 2022 (Idalino and Moura 2022), but here we provide complete proofs of these results and include a substantial number of new concepts, results and constructions.

*Our results and paper structure.* Basic concepts for cover-free families, nonadaptive group testing and hypergraphs are given in Sect. 2. The new definitions of vertex-identifying CFFs and edge-identifying CFFs are given in Sect. 3, along with related decoding algorithms. CFF constructions for hypergraphs with non-overlapping (disjoint) edges are given in Sect. 4. Constructions for the case of hypergraphs with overlapping edges are given in Sect. 5. They are parametrized using the maximum size *r* of a *defect cover*, as defined there. We give constructions for both $r=1$ and $r>1$ using edge-colouring and strong edge-colouring of hypergraphs, respectively, to partition the hypergraph into non-overlapping subgraphs that can be constructed using results from the previous section. We also give a construction using strong vertex colouring of hypergraphs for $r\ge 1$; this construction is effective when applied to consecutive hypercube hypergraphs discussed in Sect. 5.3. Swiss-army-knife CFFs are proposed in Sect. 6, as a way to use classical CFF constructions to accomplish more, using information about the underlying hypergraph. Conclusions and future work are discussed in Sect. 7.

## Preliminaries

### Cover-Free Families

Throughout the paper, we use [*a*, *b*] to denote the set $\{a,\ldots ,b\}$.

A set system $\mathcal {F} = (X, \mathcal {B})$ consists of a set *X* and a collection $\mathcal {B}$ of subsets of *X*. The *incidence matrix* *M* of the set system ${\mathcal {F}}$ is a binary matrix with rows corresponding to elements in *X* and columns corresponding to sets in $\mathcal {B}$ and such that $M_{i,j}=1$ if and only if element $x_i \in B_j$, $1\le i \le |X|$, $1\le j \le |\mathcal {B}|$.

CFFs were first introduced by Kautz and Singleton (1964) in the context of *superimposed codes*. They are equivalent to *d-disjunct matrices* and *strongly selective families* (Du and Hwang 2000; Porat and Rothschild 2011). We can define a *d*-CFF as a set system or, equivalently, as its incidence matrix. Recall that a *d*-CFF is a set system where no subset is contained in the union of *d* other subsets, which is formally defined next.

#### Definition 2.1

*(cover-free family)* Let *d*, *n*, *t* be non-negative integers with $d<n$. A *d*-cover-free family, denoted *d*-CFF(*t*, *n*), is a set system $\mathcal {F} = (X, \mathcal {B})$ with $|X| = t$ and $|\mathcal {B}| = n$ such that for any $d+1$ distinct subsets $B_{i_0}, B_{i_1}, \ldots , B_{i_d} \in \mathcal {B}$, we have

1 $$\begin{aligned} \left| B_{i_0} \setminus \bigcup _{j=1}^{d}B_{i_j}\right| \ge 1. \end{aligned}$$

Equivalently, we can define a *d*-CFF(*t*, *n*) *matrix* as the incidence matrix of the set system defined in Definition 2.1, which is a $t \times n$ binary matrix such that any set of $d+1$ columns contain a permutation submatrix of order $d+1$. For any given *n* and *d*, we are interested in *d*-CFFs with the smallest possible *t*, so we define $t(d,n) = \text {min}\{t: \exists \ d\text {-CFF}(t,n)\}$. A 1-CFF is equivalent to a Sperner system, that is, a set system where no set is contained in any other. Thus, Sperner theorem (Sperner 1928) implies

2 $$\begin{aligned} t(1,n)=\min \left\{ s: {s \atopwithdelims (){\lfloor s/2\rfloor }} \ge n\right\} . \end{aligned}$$

We note that *t*(1, *n*) grows as $\log _2 n$ as $n \rightarrow \infty$. For $d \ge 2$, there exists constants $c_1$ and $c_2$ such that

$$c_1 \frac{d^2}{\log d} \log n \le t(d,n) \le c_2 d^2 \log n.$$

The constant $c_1$ for the lower bound is shown to be $\approx 1/2, 1/4, 1/8$  (D’yachkov and Rykov 1982; Füredi 1996; Ruszinkó 1994), respectively, where the proof in (Füredi 1996) is the simplest. For $d\ge 2$, there are several approaches for constructing *d*-CFFs using codes and combinatorial designs. Probabilistic methods usually provide the best asymptotic existence results, and derandomization techniques yield polynomial time algorithms to construct a *d*-CFF(*n*, *t*) with $t = \varTheta (d^2 \log n)$ (Bshouty 2015; Gargano et al. 2018, 2020; Porat and Rothschild 2011). However, for practical values of *n* found in applications, constructions using codes (such as the one in Proposition 6.2) can yield smaller values of *t* (Demczyk 2025). For a comprehensive survey on CFFs, see Idalino and Moura (2024).

### Combinatorial group testing using CFFs

**Fig. 1:**
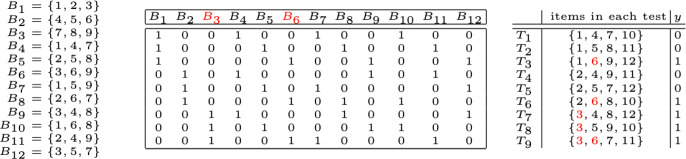
A 2-CFF(9, 12), its incidence matrix and group testing with defective $B_3$ and $B_6$. Adapted from Aldridge et al. (2019) (Color figure online)

Let us turn our attention to the non-adaptive combinatorial group testing problem discussed in Sect. 1. Consider a $t\times n$ binary matrix *M* where each test $T_i$ corresponds to a row, each item corresponds to a column *j*, and $M_{i,j}=1$ if $j\in T_i$. Let $x\in \{0,1\}^n$ be a binary vector indicating the defective items with a 1. The vector $y=Mx \in \{0,1\}^t$ is called the *vector of test outcomes*. The adaptive group testing problem with exactly *d* (at most *d*) defective items can be solved using a matrix *M* if any $x\in \{0,1\}^n$ with weight exactly *d* (at most *d*, respectively) can be determined from the vector *y* of test outcomes. A necessary and sufficient condition for a matrix *M* to solve such a problem is that *M* is *d*-separable ($\overline{d}$-separable) under the assumption of exactly *d* defectives (at most *d* defectives, respectively), as defined next.

#### Definition 2.2

(Du and Hwang (2000)) Let *d* be a positive integer. A *d*-*separable* matrix is a binary matrix where no two sets of *d* columns have the same boolean sum. A binary matrix is called $\overline{d}$-*separable* if no two sets of up to *d* columns have the same boolean sum.

Given *M*, determining *x* from *y* is called the *decoding problem* for *M*. A *d*-CFF matrix given in Definition 2.1 (often called *d*-disjunct matrix) is a special type of $\overline{d}$-separable matrix for which there is an efficient algorithm for solving the decoding problem. Indeed, it is not difficult to prove that decoding using a *d*-CFF can be done by determining $D=[1,n]\setminus \cup _{\{1\le i \le t : y_i=0\}} T_i$. This can be computed in time *O*(*tn*) (Du and Hwang 2000, 2006).

In Figure 1, we show an example of a 2-CFF(9, 12), which can be used to test $n=12$ items using $t = 9$ tests to identify up to $d=2$ defective items.

After running the tests on groups of items according to the rows of a *d*-CFF matrix *M*, we can run a simple algorithm to identify the invalid items. Here we give Algorithm 1, which works for a more general setting and is used in Sect. 3. When we apply Algorithm 1 with a *d*-CFF matrix *M* and the number of defectives is indeed bounded by *d*, then after the first loop, *x* has at most *d* nonzero components. So for *d*-CFFs, the loop in line 9 can be removed and substituted by a simple check that the number of 1’s in *x* does not exceed *d*; in this case, the output will be Boolean, i.e. every component is in $\{0,1\}$, and correct. In the case of other types of matrices or when the number of defective items exceeds *d*, the algorithm classifies the items into three types of defective status: 1, 0,  and 0.5 for defective, nondefective, and unknown, respectively.

### Hypergraphs

Hypergraphs are equivalent to set systems, but, in this paper, we use the term hypergraph when we view the objects as generalizations of graphs. In this paper, with respect to group testing, hypergraphs are used to model the structure of possible sets of defective items, while set systems are used when referring to CFFs that define the testing matrices. A *hypergraph* $\mathcal {H}$ consists of a pair $(V,\mathcal {E})$ where *V* is a finite set of elements called *vertices* and $\mathcal {E}$ is a set of nonempty subsets of *V* called *edges* (or hyperedges). A vertex *v* is incident to an edge *e* if $v\in e$. Two vertices *v*, *w* are adjacent if there exists an edge *e* with $v,w \in e$. Moreover, $\mathcal {H}$ is said to be *k*-*uniform* if every edge has cardinality *k*. Graphs are 2-uniform hypergraphs. The degree of a vertex *v* is the number of edges incident to *v*.

**Algorithm 1:**
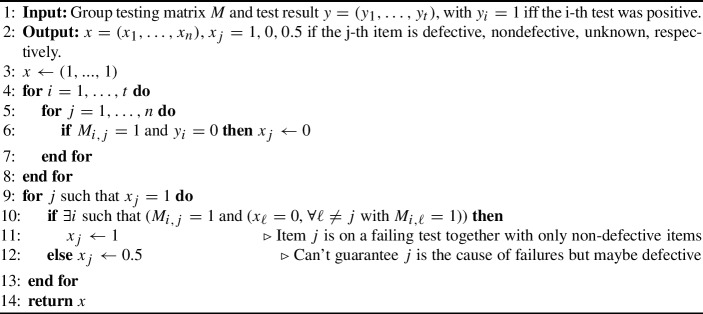
Non-adaptive CGT algorithm to identify invalid items

Let $\mathcal {H}=(V,\mathcal {E})$ be a hypergraph. An $\ell$-*vertex-colouring* of $\mathcal {H}$ is a mapping from *V* to $[1,\ell ]$ (the colours) such that no edge contains vertices all of the same colour. An $\ell$-*strong-vertex-coloring* of $\mathcal {H}$ is an $\ell$-vertex-coloring such that if two vertices belong to an edge, they must have different colours. An $\ell$-*edge-colouring* of $\mathcal {H}$ is a mapping from $\mathcal {E}$ to $[1,\ell ]$ such that no vertex is incident to more than one edge mapping to the same colour. An $\ell$-*strong-edge-coloring* of $\mathcal {H}$ is an $\ell$-edge-coloring such that any two vertices belonging to distinct edges with the same colour are not adjacent. The *chromatic number* $\chi (\mathcal {H})$ is the minimum number $\ell$ for which there exists an $\ell$-vertex-coloring. The *strong chromatic number* $\chi _s(\mathcal {H})$ is the minimum number $\ell$ for which there exists an $\ell$-strong-vertex-colouring of $\mathcal {H}$. The *chromatic index* $\chi '(\mathcal {H})$ is the minimum number $\ell$ for which there exists an $\ell$-edge-colouring of $\mathcal {H}$. The *strong chromatic index* $\chi '_s(\mathcal {H})$ is the minimum number $\ell$ for which there exists an $\ell$-strong-edge-colouring of $\mathcal {H}$. Obviously, $\chi (\mathcal {H})\le \chi _s(\mathcal {H})$ and $\chi '(\mathcal {H})\le \chi '_s(\mathcal {H})$.

## Cover-free families on hypergraphs

We consider cover-free families on an underlying hypergraph structure, where vertices correspond to items/columns of the CFF and edges specify potential clusters of defective items. When screening for an infectious disease, vertices represent people and edges represent communities of people with a high level of contact. For example, the vertices can represent students, and the edges can represent the classrooms in a school or the course sections in a university. We use the assumption that defective items are contained in a small number *r* of edges, inside of which any positive number of defective items may be present.

Other works have also considered a hypergraph model in group testing (De Bonis 2024; Gonen et al. 2022; Vorobyev 2022; Nikolopoulos et al. 2021a, b, 2023). The model in (De Bonis 2024; Gonen et al. 2022; Vorobyev 2022) focus on $r=1$, so their hypergraph would be different than ours when $r>1$; when our hypergraph has *m* edges, theirs would have ${m \atopwithdelims ()r}$ edges. In addition, they assume that edges represent the (exact) possible sets of defective items, while in our model edges contain possible sets of defective items; therefore, to model the same problem as ours, all subsets of edges must be added to their model.

From now on, we assume that every hypergraph $\mathcal {H}=(V,\mathcal {E})$ has no isolated vertices, which is realistic for the group testing application, since items that are not in an edge cannot be defective and can be directly removed from the model. With this assumption, the set $\mathcal {E}$ is enough to specify the hypergraph, as the vertex set is simply the union of all the edges.

We introduce two different types of cover-free families on hypergraphs: *vertex-identifying CFFs* and *edge-identifying CFFs*. As long as the set of defective vertices is covered by at most *r* edges, the first allows the identification of infected vertices, while the second guarantees only the identification of infected edges. The names given to these CFFs are slightly changed with respect to the earlier (conference) version of this paper (Idalino and Moura 2022).

### Definition 3.1

*(Vertex-identifying CFFs)* Let $n,t> 0$ and $r\ge 0$ be integers. Let $\mathcal {H} = ([1,n], \mathcal {E})$ be a hypergraph with *n* vertices and *m* edges, and let *M* be a $t \times n$ binary matrix with associated set system $\mathcal {F}_{M} = ([1,t], \mathcal {B})$, $\mathcal {B} = \{B_1, \ldots , B_n\}$. The matrix *M* is a *vertex-identifying cover-free family*, denoted $(\mathcal {E},r)$-CFF(*t*, *n*), if for any *r*-set of edges $\{e_1,\ldots ,e_r\}\subseteq \mathcal {E}$, and for any $I \subseteq \cup _{j=1}^r e_j$ and any $i_0\in [1,n]\setminus I$, we have

3 $$\begin{aligned} \bigg |B_{i_0} \Big \backslash \bigg (\bigcup _{i \in I}B_{i}\bigg )\bigg | \ge 1. \end{aligned}$$

A *d*-CFF(*t*, *n*) is equivalent to an $(\mathcal {E},1)$-CFF(*t*, *n*) where $\mathcal {E}$ is the set of all *d*-subsets of [1, *n*]. A *d*-CFF(*t*, *n*) is also equivalent to an $(\mathcal {E},d)$-CFF(*t*, *n*) where edges of $\mathcal {H}$ are singleton vertices: $\mathcal {E}=\{\{1\},\{2\},\ldots \{n\}\}$.

Next, we define edge-identifying CFFs, which have a weaker coverage requirement than vertex-identifying CFFs.

### Definition 3.2

*(Edge-identifying CFFs)* Let *r*, *t*, *n*, *M*, $\mathcal {H}$ and $\mathcal {F}_{M}$ be as in Definition 3.1. The matrix *M* is an $(\mathcal {E},r)$-ECFF(*t*, *n*) if for any $\ell$-subset of edges $\{e_1,\ldots ,e_\ell \} \subseteq \mathcal {E}$, $\ell \le r$, and any ${i_0}\notin E = \cup _{j=1}^{\ell } e_j$, we have

4 $$\begin{aligned} \bigg |B_{i_0} \Big \backslash \bigg (\bigcup _{i \in E}B_{i}\bigg )\bigg | \ge 1. \end{aligned}$$

Removing a column from a CFF (ECFF) for any hypergraph $\mathcal {H}$ and deleting the corresponding vertex from $\mathcal {H}$ yields a CFF (ECFF) for the modified hypergraph. In addition, adding rows to a CFF (ECFF) for any hypergraph $\mathcal {H}$ results in a CFF (ECFF) for the hypergraph.

Let *r* be a positive integer and $\mathcal {H}=([1,n],\mathcal {E})$ be a hypergraph with no isolated vertices. Throughout this article, we are interested in minimizing the number *t* of rows of the CFFs on $\mathcal {H}$, thus we use the following notation:

$$\begin{aligned} t(r,{\mathcal {E}})= &  \min \{t: \exists \ (\mathcal {E},r)\text {-CFF}(t,n)\},\\ t_E(r,{\mathcal {E}})= &  \min \{t: \exists \ (\mathcal {E},r)\text {-ECFF}(t,n)\}. \end{aligned}$$

An edge is *defective* if it contains a defective vertex and *non-defective*, otherwise. A set of edges is a *defect cover* if the set of defective vertices is contained in the union of these edges; such a defect cover is *minimal* if no proper subset is a defect cover, and it is *minimum* if has the smallest cardinality among all defect covers. A minimal defect cover is always contained in the set of defective edges, but the number of defective edges may be much larger than the size of a defect cover for hypergraphs with overlapping edges. The next proposition shows that a vertex-identifying CFF’s ability to detect defectives only depends on the cardinality of a minimum defect cover being bounded by *r*.

### Proposition 3.3

Let $\mathcal {H} = ([1,n], \mathcal {E})$ be a hypergraph, *M* be an $(\mathcal {E},r)$-CFF(*t*, *n*) and $y\in \{0,1\}^t$ be the result of tests given by *M* on items $1,\ldots , n$. If $\mathcal {H}$ has a defect cover with at most *r* edges, then Algorithm 1 on inputs (*M*, *y*) returns a Boolean output *x* such that $x_i=1$ if and only if item *i* is defective.

### Proof

Let $\mathcal{D}\mathcal{C}=\{e_1,e_2,\ldots ,e_{\ell }\}$ be a defect cover with $\ell \le r$. Let ${i_0}\in [1,n]$ be an item and take $I=(\cup _{i=1}^{\ell } e_i)\setminus \{{{i_0}}\}$. Since *M* is an $(\mathcal {E},r)$-CFF(*t*, *n*), Equation (3) guarantees there exists a row *w* in *M* that has a 1 in column ${i_0}$ and has a 0 in all columns in *I*. If item ${{i_0}}$ is non-defective, this row will be a passing test, $y_w=0$, and $x_{i_0}$ will be set to 0 in the first loop. Otherwise, item ${i_0}$ is defective, and $x_{i_0}$ will remain equal to 1 at the end of the first loop. In addition, row *w* will prove that the condition on the second loop is true for $i=i_0$, so $x_{i_0}$ will be set to 1. Therefore, the output of Algorithm 1 will be a Boolean *x* that correctly informs the status of the items. $\square$

We are also interested in identifying defective edges when the output of Algorithm 1 is not Boolean, which can happen if defective items are spread over too many edges (any defect cover has size $>r$) or the matrix used is an ECFF rather than a CFF. For example, in schools, the tests may not provide full information on infected students, but we still may extract information on which classrooms are infected. Algorithm 2 provides edge information based on ternary vertex information for a hypergraph ${\mathcal {H}}$. The first loop searches over all vertices of each edge and sets the respective position of vector *z* to 1 if the edge has at least one infected vertex. The last loop considers a set *C*, which contains the defective items responsible for the failure of the *i*-th test ($y_i=1$). Then, if *C* is contained in an edge, this edge must be defective.

**Algorithm 2:**
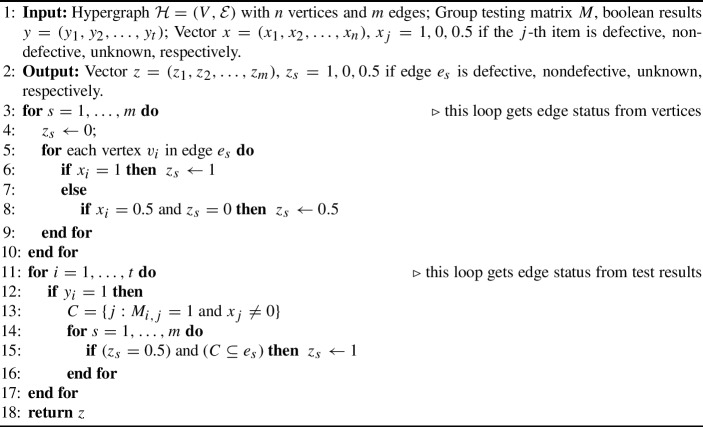
Edge information from vertices

Some CFFs may have a value of *r* for vertex status identification but have a larger value of *r* for edge status identification (see, for example, Proposition 6.1 and Theorem 6.3). This can be useful for applications, in that infected communities are identifiable even though we do not have perfect individual identification.

### Proposition 3.4

Let $\mathcal {H} = ([1,n], \mathcal {E})$ be a hypergraph, let *M* be an $(\mathcal {E},r)$-ECFF(*t*, *n*), and let *y* be the test results for *M*. Let *x* be the output of Algorithm 1 for inputs $(\mathcal {H},M,$y). Then, if $\mathcal {H}$ has a defect cover with at most *r* edges then Algorithm 2 applied to $(\mathcal {H}, M,$x, y) returns an output *z* such that $\{e_s\in \mathcal {E}: z_s=1\}$ forms a defect cover that only contains defective edges.

### Proof

Let $\mathcal{I}\mathcal{C}=\{e_{i_1},\ldots ,e_{i_\ell }\}$ be any minimal defect cover with $\ell \le r$ and let $E=\cup _{j=1}^{\ell } e_{i_j}$. Then, any item ${i_0}\not \in E$ is non-defective and Equation (4) guarantees there is a row *w* that tests ${i_0}$ and avoids all items in *E*, and thus avoids all defective items, which means $y_w=0$ and Algorithm 1 sets $x_{{i_0}}=0$. Now, consider any edge $e_s \in \mathcal{I}\mathcal{C}$ and let $S=\bigcup _{X\in \mathcal{I}\mathcal{C}\setminus \{e_s\}} X$. Since $\mathcal{I}\mathcal{C}$ is minimal, the edge $e_s$ must contain a defective item $u \in e_s\setminus S$. By Equation (4), using $i_0=u$, there must be a test/row *w* that contains *u* and avoids *S*. Thus, we must have $\{j: M_{w,j}=1\ \textrm{and}\ x_j \not =0\} \subseteq e_s$, which implies Algorithm 2 sets $z_s=1$ in the second loop. Therefore, $z_{i} =1$ for all ${e_i}\in \mathcal{I}\mathcal{C}$ and possibly for a few other edges. Since every superset of a defect cover is a defect cover, we have that $\{e_i \in \mathcal {E}: z_i=1\}$ is a defect cover. Note that even in this case, *z* does not have to be Boolean. $\square$

### Proposition 3.5

Let $\mathcal {H} = ([1,n], \mathcal {E})$ be a hypergraph with $|E| = m$, let *M* be an $(\mathcal {E},r)$-ECFF(*t*, *n*), and *y* be the test results for *M*. Let *x* be the output of Algorithm 1 for inputs $(\mathcal {H},M,$y), and let *z* be the output of Algorithm 2 applied to $(\mathcal {H}, M,$x, y). Let $D = \{e_s\in \mathcal {E}: z_s=1\}$. Then, if $\mathcal {H}$ has a defect cover with at most *r* edges, such defect cover can be identified in time $O(n \cdot (|D|+1)^r) = O(n \cdot (m+1)^r)$.

### Proof

By Proposition 3.4, we have that *D* is a defect cover. For each nonempty subset of edges $S \subseteq D$, check whether it is a defect cover as follows. We just need to check that each item $j \in [1,n] \setminus \cup _{e \in S} e$ has $x_j = 0$ to conclude that *S* is a defect cover. This check can be done in *O*(*n*) and there are $\sum _{i=1}^r {|D| \atopwithdelims ()i} \le (|D|+1)^r= O((|D|+1)^r)$ such subsets *S* to check. $\square$

Now we define an *intra-edge-screening* CFF. This object represents which extra properties need to be added to an ($\mathcal {E}, r$)-ECFF to obtain an ($\mathcal {E}, r$)-CFF.

### Definition 3.6

*(Intra-edge-screening CFFs)* Let *r*, *t*, *n*, *M*, $\mathcal {H}$ and $\mathcal {F}_{M}$ be as in Definition 3.1. We say *M* is an $(\mathcal {E},r)$-InCFF(*t*, *n*) if for any *r*-set of edges $\{e_1,\ldots ,e_r\}\subseteq \mathcal {E}$, and for any $I \subset \cup _{j=1}^r e_j$ and any $i_0 \in \cup _{j=1}^r e_j \setminus I$, we have

5 $$\begin{aligned} \bigg |B_{i_0} \Big \backslash \bigg (\bigcup _{i \in I}B_{i}\bigg )\bigg | \ge 1. \end{aligned}$$

In this case, we use the following notation:

$$\begin{aligned} t_{in}(r,\mathcal {E})= &  \min \{t: \exists \ (\mathcal {E},r)\text {-InCFF}(t,n)\}. \end{aligned}$$

### Proposition 3.7

Let $\mathcal {H} = ([1,n], \mathcal {E})$ be a hypergraph. Then *M* is an $(\mathcal {E},r)$-CFF(*t*, *n*) if and only if *M* is an $(\mathcal {E},r)$-ECFF(*t*, *n*) and is an $(\mathcal {E},r)$-InCFF(*t*, *n*).

### Proof

Let $E = \{e_1, \ldots , e_{\ell }\}\subseteq \mathcal {E}$, $I \subseteq \cup _{j=1}^{\ell } e_j$, for $\ell \le r$. We know that *M* is an $(\mathcal {E},r)$-CFF(*t*, *n*) if and only if for any such choice of *E* and *I* and for any $i_0 \in [1,n]\setminus I$, there exists a row *p* such that $M_{p,i_0}=1$ and $M_{p,i}=0$ for all $i \in I$. This is true if and only if for any such choice of *E* and *I*, the following are true:

- For any $i_0 \in [1,n] \setminus \cup _{j=1}^{\ell } e_j$, there exists a row *p* such that $M_{p,i_0}=1$ and $M_{p,i}=0$ for all $i \in I$; and
- For any $i_0 \in \cup _{j=1}^{\ell } e_j \setminus I$ there exists a row *p* such that $M_{p,i_0}=1$ and $M_{p,i}=0$ for all $i \in I$.

This is equivalent to *M* being an $(\mathcal {E},r)$-ECFF(*t*, *n*) (see Equation (5)) and *M* being an $(\mathcal {E},r)$-InCFF(*t*, *n*) (see Equation (3)). $\square$

The *vertical concatenation* of matrices *A* and *B* (with the same number of columns) is the matrix consisting of rows of *A* followed by the rows of *B*. Proposition 3.7 implies a method for building CFFs from vertical concatenation of ECFFs and InCFFs, given in the next proposition.

### Proposition 3.8

Let $\mathcal {H} = ([1,n], \mathcal {E})$ be a hypergraph. If *M* is an $(\mathcal {E},r)$-ECFF($t_1,n$) and *N* is an $(\mathcal {E},r)$-InCFF($t_2,n$), then the vertical concatenation of matrices *M* and *N* is an $(\mathcal {E},r)$-CFF($t_1+ t_2,n$).

The following bounds are immediate from Propositions 3.7 and 3.8.

### Corollary 3.9

Let $\mathcal {H} = ([1,n], \mathcal {E})$ be a hypergraph. Then,

$$ \max \{ t_{E}(r,\mathcal {E}),t_{in}(r,\mathcal {E})\} \le t(r,\mathcal {E}) \le t_{E}(r,\mathcal {E})+t_{in}(r,\mathcal {E}).$$

## Cover-free families on hypergraphs: non-overlapping edges

Here we consider the case of non-overlapping edges, meaning that items do not participate in more than one edge. In this case, we assume without loss of generality that the vertex set is [1, *n*] and that the vertices appear in their natural order in edges $e_1, e_2, \ldots , e_m$. For example, if there are *m* edges of cardinality *k* then $n=mk$, $e_1=[1,k], e_2=[k+1,2k], \ldots , e_m=[(m-1)k+1,mk]$.

Let $A_k$ be a $p_k \times n_k$ binary matrix, for $k=1,2$, and **0** be the matrix of all zeroes with the same dimension as $A_2$. The Kronecker product $P = A_1 \otimes A_2$ is a $p_1p_2 \times n_1n_2$ binary matrix formed of blocks *P*(*i*, *j*) such that $P(i,j) = A_2$ if $A_{1_{i,j}} = 1$ and $P(i,j) = {\textbf {0}}$, otherwise. We denote by $R_k$ the row matrix with *k* ones and by $I_k$ the identity matrix of dimension *k*.

### Proposition 4.1

Let $\mathcal {H}=([1,n],\mathcal {E})$ be a hypergraph formed by *m* disjoint edges of cardinality *k*, $n=mk$. Let *A* be an *r*-CFF(*t*, *m*). Then $A\otimes R_k$ is an $(\mathcal {E},r)$-ECFF(*t*, *mk*). In addition,

6 $$\begin{aligned} t_E(r,\mathcal {E}) = t(r,m). \end{aligned}$$

### Proof

Let $\{e_{j_1},\ldots ,e_{j_{\ell }}\} \subseteq {\mathcal {E}}$, $\ell \le r$, let $E=\cup _{u=1}^\ell e_{j_u}$, and let $i_0 \in [1,n] \setminus E$. Thus $i_0 \in e_{j_0}$, where $j_0=\lceil i_0/k\rceil$. The array $B=A\otimes R_k$ has *m* column blocks of *k* columns each. Because *A* is an *r*-CFF, there exists a row *s* such that $A_{s,j_1}=A_{s, j_2}=\cdots =A_{s,j_{\ell }}=0$ and $A_{s,j_0}=1$. Thus, in *B*, we have $B_{s,i}=0$ for all $i \in E$ and $B_{s,i_0}=1$. Therefore, Equation (4) is satisfied, and *B* is an $(\mathcal {E},r)$-ECFF(*t*, *mk*). This yields $t_E(r,\mathcal {E}) \le t(r,m)$.

We now prove the lower bound, $t(r,m)\le t_E(r,\mathcal {E})$. We can observe that for hypergraphs with non-overlapping edges, it is evident that finding a defective cover of size at most *r* among *m* edges using an ECFF (for a hypergraph with non-overlapping edges) is equivalent to solving an ordinary group testing for *r* defective items among *m* items using an *r*-CFF(*t*, *m*). Indeed, for any ECFF with *t* rows, we can assume without loss of generality that all entries in any row have the same value (either 0 or 1) for all vertices corresponding to an edge. Indeed, if a row contains a mix of 0s and 1s for positions corresponding to vertices of an edge, we can change all these values to 1, without violating Equation (4). Then, by keeping one column corresponding to a vertex in each edge yields an *r*-CFF(*t*, *m*). Thus, $t_E(r,\mathcal {E}) = t(r,m)$. $\square$

It is worth noting that Proposition 4.1 also holds for hypergraphs with non-overlappings edges of different sizes.

### Proposition 4.2

Let $\mathcal {H}=([1,n],\mathcal {E})$ be a hypergraph formed by *m* disjoint edges of cardinality *k*, $n=mk$. Let *B* be an $(r-1)$-CFF(*t*, *m*). Then $B\otimes I_k$ is an $(\mathcal {E},r)$-InCFF(*tk*, *mk*). This implies $t_{in}(r,\mathcal {E}) \le t(r-1,m)\times k$.

### Proof

Note that $C=B\otimes I_k$ has *m* “column blocks” with *k* columns each and *t* “row blocks” with *k* rows each. Let $\{e_{j_1},e_{j_2},\ldots , e_{j_{r}}\} \subseteq \mathcal {E}$. Let $E=\cup _{u=1}^r e_{j_u}$, $I\subset E$ and $i_0\in E \setminus I$. Let $\ell \in [1,r]$ such that $i_0\in e_{j_{\ell }}$ is the *p*th vertex of edge $e_{j_{\ell }}$. Since *B* is an $(r-1)$-CFF, there exists a row $1\le s\le t$ such that $B_{s,j_{\ell }}=1$ and $B_{s,j_u}=0$, for all $1\le u \le r$, $u\not =\ell$. Thus, the corresponding blocks of *C* are $C(s,j_{\ell })=I_k$ and $C(s,j_u)={\textbf {0}}$, for all $1\le u \le r$, $u\not =\ell$. The *p*-th row across these blocks will have a 1 in the *p*th column of block $C(s,j_{\ell })$ and 0 in all other columns. This implies, noting that $i_0=(j_{\ell }-1)k+p$ and taking $s'=(s-1)k+p$, that $C_{s',i_0}=1$ and $C_{s',i}=0$ for all $i\in I$. Therefore, Equation (5) is satisfied. Thus, *C* is an $(\mathcal {E},r)$-InCFF(*tk*, *mk*). $\square$

The next result is inspired by the following recursive construction of *d*-CFFs. Let $A_1$ be a *d*-CFF$(t_1, n_1)$ and $A_2$ be a *d*-CFF$(t_2, n_2)$, then $C = A_1 \otimes A_2$ is a *d*-CFF$(t_1t_2, n_1 n_2)$ (Idalino and Moura 2021).

### Theorem 4.3

Let $\mathcal {H}=([1,n],\mathcal {E})$ be a hypergraph formed by *m* disjoint edges of cardinality *k*, $n=mk$. Let *r* be a positive integer, and let *A* be an *r*-CFF(*t*, *m*). Then $A\otimes I_k$ is an $(\mathcal {E},r)$-CFF(*tk*, *n*). Moreover, if edges have different cardinalities $k_1, \ldots , k_m$ bounded by *k*, a similar construction yields an $(\mathcal {E},r)$-CFF$(tk,n = \sum _{i=1}^m k_i)$. This yields $t(r,\mathcal {E}) \le t(r,m) \times k$.

### Proof

Let us first consider the uniform case, where $\mathcal {H}$ has *m* disjoint edges of cardinality *k*. Since *A* is an *r*-CFF, we have that *A* is an $(r-1)$-CFF, so by Proposition 4.2, $A\otimes I_k$ is an $(\mathcal {E},r)$-InCFF(*tk*, *mk*). By Proposition 4.1, $A\otimes R_k$ is an $(\mathcal {E},r)$-ECFF(*t*, *mk*). We claim that this implies $A\otimes I_k$ is an $(\mathcal {E},r)$-ECFF(*tk*, *mk*). Indeed one just need to observe that each row of $A\otimes R_k$ is a logical-or of the rows in each corresponding “row block” of $A\otimes I_k$; alternatively, one can follow the proof of Proposition 4.1 and see that the same argument used for $A\otimes R_k$ applies to $A\otimes I_k$. Therefore, since $C=A\otimes I_k$ is an $(\mathcal {E},r)$-InCFF(*tk*, *mk*) and an $(\mathcal {E},r)$-ECFF(*tk*, *mk*), by Proposition 3.7, *C* is an $(\mathcal {E},r)$-CFF$(tk,n=mk)$.

For the case of non-uniform hypergraphs, where each edge $e_i$ has cardinality $k_i \le k$, we follow a similar construction. We build $A\otimes I_k$, but for each block *i*, corresponding to edge $e_i$, remove any $k-k_i$ extra columns. The resulting matrix *D* has the same number *tk* of rows as *C*, $n = \sum _{i=1}^{m}k_i$ columns, and by similar argments as the uniform case, *D* is an $(\mathcal {E},r)$-CFF$(tk,n = \sum _{i=1}^m k_i)$. $\square$

The next result is inspired by the following recursive construction of *d*-CFFs: if $A_1$ is a *d*-CFF$(t_1, n_1)$, $A_2$ is a *d*-CFF$(t_2, n_2)$ and *B* is a $(d-1)$-CFF$(s, n_2)$, then the vertical concatenation of $B\otimes A_1$ and $A_2 \otimes R_{n_1}$ is a *d*-CFF$(st_1 + t_2, n_1 n_2)$ (Idalino and Moura 2021).

### Theorem 4.4

Let $\mathcal {H}=([1,n],\mathcal {E})$ be a hypergraph formed by *m* disjoint edges of cardinality *k*, $n=mk$. Let *r* be a positive integer, *A* be an *r*-CFF$(t_A,m)$, and *B* be an $(r-1)$-CFF$(t_B,m)$. Then the vertical concatenation of $A\otimes R_k$ and $B\otimes I_k$ is an $(\mathcal {E},r)$-CFF$(t_A+ t_B k,n)$. Moreover, if edges have different cardinalities $k_1, \ldots , k_m$ bounded by *k*, a similar construction yields an $(\mathcal {E},r)$-CFF$(t_A+ t_B k,n = \sum _{i=1}^m k_i)$. This yields $t(r,\mathcal {E}) \le t(r,m) + t(r-1,m)\times k$.

### Proof

By Proposition 4.1, $M = A\otimes R_k$ is an $(\mathcal {E},r)$-ECFF$(t_A,mk)$ and, by Proposition 4.2, $N = B\otimes I_k$ is $(\mathcal {E},r)$-InCFF$(t_Bk,mk)$. Consequently, by Proposition 3.8, their vertical concatenation is an $(\mathcal {E},r)$-CFF$(t_A+ t_B k,n)$. For the case of non-uniform hypergraphs, where each edge $e_i$ has cardinality $k_i \le k$, we first use the same construction by vertically concatenating *M* and *N* to form *C*. This construction builds matrices formed by *m* column blocks (from the CFFs) of *k* columns each (from $I_k$ and $R_k$). For each block *i* of *C*, corresponding to edge $e_i$, we remove any $k-k_i$ extra columns, and form *D*. The resulting matrix *D* has the same number $t_A + t_B k$ of rows as *C*, but $n = \sum _{i=1}^{m}k_i$ of columns, and by similar arguments as the uniform case, *D* is an $(\mathcal {E},r)$-CFF$(t_A+ t_B k, n = \sum _{i=1}^m k_i)$. $\square$

**Fig. 2:**
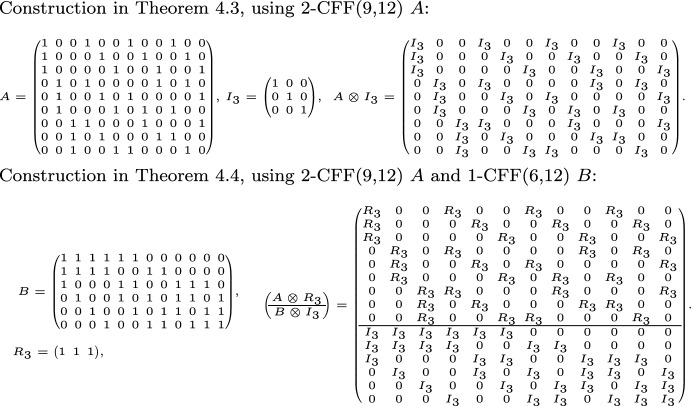
Two $(\mathcal {E},2)$-CFF(27, 36), the set of edges $\mathcal {E}$ of hypergraph $\mathcal {H}$ consists of 12 disjoint edges of size 3. Up to six defective items concentrated within 2 edges can be identified

Figure 2 shows examples of CFFs using the constructions in Theorems 4.3 and 4.4.

As a consequence of Theorem 4.4 for the case $r=1$, we can construct an $(\mathcal {E},1)$-CFF from a 1-CFF on edges and a horizontal concatenation of identity matrices. Corollary 4.5 specifies this important special case where we can obtain explicit upper bounds.

### Corollary 4.5

($r=1$) Let $\mathcal {H}=([1,n],\mathcal {E})$ be a hypergraph formed by *m* disjoint edges of cardinality *k*, $n=mk$. Then

$$\begin{aligned} t_E(1,\mathcal {E})= &  t(1,m)=\min \{s: {s \atopwithdelims (){\lfloor s/2\rfloor }} \le m\}=\varTheta (\log m),\\ t(1,\mathcal {E})\le &  t(1,m)+ k = \varTheta (\log m +k). \end{aligned}$$

Moreover, the same bounds hold if the *m* disjoint edges have possibly different cardinalities $k_1, \ldots , k_m$ bounded by *k*.

### Proof

Let *A* be a 1-CFF$(t',m)$ matrix, where $t'$ is the smallest possible number of rows when fixing *m* columns. Sperner’s theorem implies $t'=t(1,m)=\min \{s: {s \atopwithdelims (){\lfloor s/2\rfloor }} \le m\}$. By Stirling’s approximation, we know $t(1,m)=\varTheta (\log m)$. Let $M=A \otimes R_k$, by Proposition 4.1, *M* is an $(\mathcal {E},1)$-ECFF$(t',n)$. By Equation 6, $t_E(1,\mathcal {E}) = t(1,m)$, which concludes the first line of equalities. Let $N=R_m \otimes I_k$, noting that $R_m$ is a 0-CFF, by the proof of Theorem 4.4 using $B=R_m$, we conclude that *N* is an $(\mathcal {E},1)$-InCFF(*k*, *n*), and the vertical concatenation of *M* and *N* is an $(\mathcal {E},1)$-CFF$(t'+k,n)$, implying the second line of inequalities. Moreover, for the case of non-uniform hypergraphs, where each edge $e_i$ has cardinality $k_i \le k$, we use the same strategy as in Theorem 4.4: remove any $k-k_i$ extra columns for the block of columns corresponding to each $e_i$.

$$\square $$

Consider the bounds for $t_E(r,\mathcal {E})$ and $t(r,\mathcal {E})$ given by the propositions and theorems above, namely, $t_E(r,\mathcal {E}) = t(r,m)$ (Proposition 4.1), $t(r,\mathcal {E}) \le t(r,m) \times k$ (Theorem 4.3), $t(1,\mathcal {E}) \le t(1,m)+ k$ (Corollary 4.5), and $t_E(r,\mathcal {E})\le t(r,\mathcal {E})$ (Corollary 3.9). Consider also the known lower bound for classical CFFs: $t(d,n) \ge c\frac{d^2}{\log d } \log n$ for some constant *c* (Füredi 1996; Ruszinkó 1994; Wei 2006), $t(1,n) \ge \log _2 n$, and the upper bound $\varTheta (d^2 \log n)$ (Bshouty 2015; Gargano et al. 2018, 2020; Porat and Rothschild 2011). We combine these results in Table 1 to give lower and upper bounds for $t_E(r,\mathcal {E})$ and $t(r,\mathcal {E})$ for disjoint non-overlapping edges. It is easy to observe the improvements on bounds for CFFs over hypergraphs, when compared with traditional CFFs.

**Table 1:** We consider a hypergraph $\mathcal {H} = (\mathcal {E}, V)$ with $|\mathcal {E}| = m$, and non-overlapping edges where each edge has constant size *k*. In other words, $n = mk$

CFF	Lower Bound	Upper Bound
$(\mathcal {E},1)$-ECFF(*t*, *n*)	$\log (n/k)$	$\log (n/k)$
$(\mathcal {E},1)$-CFF(*t*, *n*)	$\max \{\log (n/k),k\}$	$\log (n/k) +k$
$(\mathcal {E},r)$-ECFF(*t*, *n*)	$c_1\frac{r^2 \log (n/k)}{\log r}$	$c_2\cdot r^2 \log (n/k)$
$(\mathcal {E},r)$-CFF(*t*, *n*)	$c_1\frac{r^2 \log (n/k)}{\log r}$	$c_3\cdot k \cdot r^2 \log (n/k)$

## Cover-free families on hypergraphs: overlapping edges

Here, we consider the general case of hypergraphs where edges may overlap. The case of defect cover of size $r=1$ is considered by several authors (De Bonis 2024; Gonen et al. 2022; Vorobyev 2022), as they define edges as the sets of possible defective elements. Our approach is to define edges as some sort of “clusters” where defective elements may lie, allowing more than one cluster to contain defectives ($r\ge 1$); this alines with the model of Nikolopoulos et al. (2021a, 2021b, 2023). The case of disjoint edges considered in Sect. 4 is much simpler, but the problem becomes more interesting when we have overlapping edges.

The first constructions in this section use edge colouring of hypergraphs to partition the edges of the graph into sets of non-overlapping edges (colour classes), allowing the use of constructions from Sect. 4 to deal with each colour class. When $r=1$, we can use edge colouring, and when $r>1$, we need to use strong edge colouring. In addition, we provide a construction that uses strong vertex colouring for $r\ge 1$. In particular, this construction is applied to a hypergraph that models “consecutive defectives” on a hypercube.

### Hypergraphs with overlapping edges and defect cover of size $r=1$

#### Theorem 5.1

(Overlapping edges, $r=1$) Let $\mathcal {H}=([1,n],\mathcal {E})$ be a hypergraph and let ${\mathcal C}_1, \mathcal {C}_2, \ldots , \mathcal {C}_\ell$ be the sets of edges in each colour class of an $\ell$-edge-colouring of $\mathcal {H}$. For each *i*, $1\le i \le \ell$, let $k_i=\max \{|e|:e\in \mathcal {C}_i\}$ and let $c_i=|\mathcal {C}_i|$, Then, if there exist 1-CFF$(t_i,c_i)$ for each $1\le i \le \ell$, then there exist an $(\mathcal {E},1)$-CFF(*t*, *n*) where $t=\sum _{i=1}^\ell (t_i + k_i)$, and an $(\mathcal {E},1)$-ECFF$(\sum _{i=1}^\ell t_i,n)$.

#### Proof

Let $V_i=\cup _{e \in \mathcal {C}_i} e$ and $X_i=[1,n]\setminus V_i$, for each $1\le i \le \ell$. Let $\mathcal {H'}_i=(V_i,\mathcal {C}_i)$. Since $\mathcal {H'}_i$ is a hypergraph with non-overlapping edges, the construction in the proof of Corollary 4.5 gives a $( \mathcal {C}_i,1)$-ECFF$(t_i,|V_i|)$ $M'_i$ and and a $(\mathcal {C}_i,1)$-InCFF$(k_i,|V_i|)$ $N'_i$. Let $\mathcal {H}_i=([1,n],\mathcal {C}_i)$, which has the same edges of $\mathcal {H}'_i$ without removing the isolated vertices in $X_i$. Extend $M'_i$ to matrix $M_i$ by adding one column of 1s for each vertex in $X_i$; similarly, extend $N'_i$ to matrix $N_i$ by adding one column of 0s for each vertex in $X_i$. It is easy to see that $M_i$ is an ECFF for $\mathcal {H}_i$ and $N_i$ is an InCFF for $\mathcal {H}_i$, since the addition of columns of 1s (0’s respectively) deals appropriately with the isolated vertices.

Now, we vertically concatenate all arrays $M_1, M_2, \ldots , M_\ell$ to form a $(\sum _i^\ell t_i)\times n$ matrix *M* and all $N_1, N_2, \ldots , N_\ell$ to form a $(\sum _i^\ell k_i)\times n$ array *N*. We claim that *M* is an $(\mathcal {E},1)$-ECFF$(\sum _{i=1}^\ell t_i,n)$ and *N* is an $(\mathcal {E},1)$-InCFF$(\sum _{i=1}^\ell k_i,n)$. Indeed, let $e\in \mathcal {E}$ be an arbitrary edge, we know *e* must be in some color class $\mathcal {C}_i$. For any $i_0\in [1,n]\setminus e$, $M_i$ guarantees the existence of a row satisfying Equation 4. Thus *M* is an $(\mathcal {E},1)$-ECFF$(\sum _{i=1}^\ell t_i,n)$. In addition, for any $i_0 \in e$, $N_i$ guarantees the existence of a row satisfying Equation 5. Thus *N* is an $(\mathcal {E},1)$-InCFF$(\sum _{i=1}^\ell k_i,n)$. Finally, by Proposition 3.8, the vertical concatenation of *M* and *N* is a $(\mathcal {E},1)$-CFF(*t*, *n*). $\square$

#### Corollary 5.2

Let $\mathcal {H}=([1,n],\mathcal {E})$ be a *k*-uniform hypergraph. Then

$$\begin{aligned} t_E(1,\mathcal {E})\le &  \chi '(\mathcal {H})\cdot t(1,\lfloor (n/k) \rfloor ) \sim \chi '(\mathcal {H})\cdot \log (n/k),\\ t(1,\mathcal {E})\le &  \chi '(\mathcal {H})\cdot (t(1,\lfloor (n/k)\rfloor )+k) \sim \chi '(\mathcal {H}) \cdot ((\log (n/k)) + k). \end{aligned}$$

#### Proof

We can apply Theorem 5.1 with $\ell =\chi '(\mathcal {H} )$, $k_i=k$ for all $1\le i\le \ell$ and note that each colour class contains at most $\lfloor n/k \rfloor$ edges. The bounds for the CFFs for the individual colour classes is explicit in Corollary 4.5. $\square$

#### Example 1

**Fig. 3:**
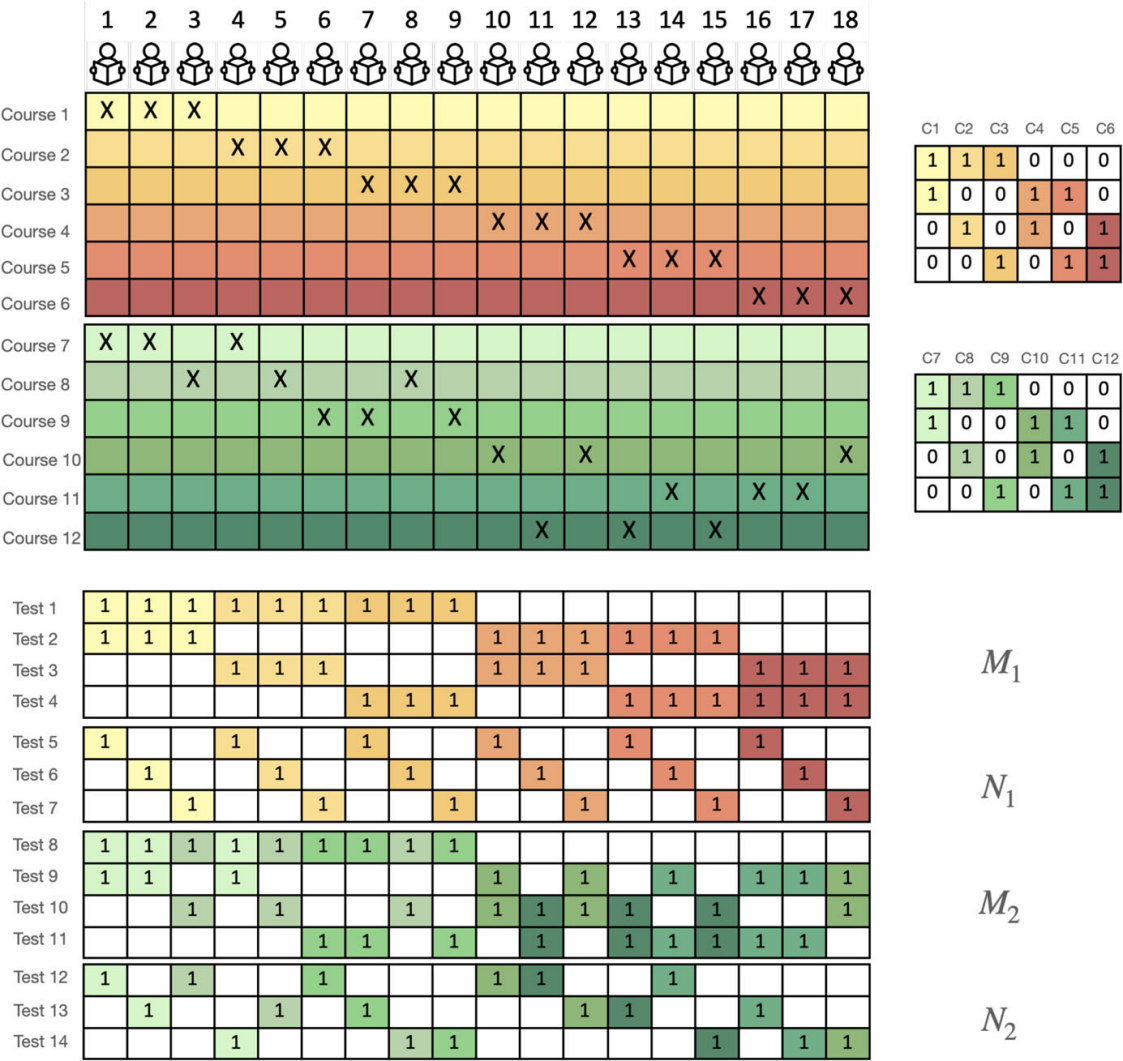
Construction of an $(\mathcal {E},1)$-CFF(14, 18) generated from Theorem 5.1

**Outbreak in high school - toy example.** In this example, we consider an outbreak of a contagious disease in a high school. Consider a high school where each student takes *P* courses per term, in *P* weekly time periods where in each time period each student attends one course of their choice. Consider a hypergraph with *n* vertices corresponding to students and each edge corresponding to students in a course. In this example, $\ell =P$, since each time period forms a colour class. We give a toy example, with $n=18$ students spread over $P = 2$ time periods (morning/afternoon), each with 6 optional courses with 3 students per course. This hypergraph has $m=12$ edges and $n=18$ vertices as shown in Figure 3. The top of the figure shows how students (vertices) are spread into courses (edges) and how the set of edges is partitioned into two colour classes, say orange (courses 1-6) and green (courses 7-12). The bottom of the figure shows the $(\mathcal {E},1)$-CFF(14, 18) generated from Theorem 5.1. The 1-CFF(4, 6) used in the construction is presented on the right side of the figure. In this case, we needed two of them (one for each colour class). The submatrices that represent the ECFFs and the InCFFs are indicated with $M_i$ and $N_i$, respectively. Our assumption is that all infected students belong to the same course (edge). For example, if students 1 and 4 are sick, course 7 is a defect cover of size $r=1$. Using tests 1-4 and 8-11 as the ECFF will cause Algorithm 2 to output courses 1, 2 and 7 as containing infected students. Moreover, using the method in Proposition 3.5, we can find that course 7 is a defect cover of size 1, i.e., a course where the outbreak occurred. Alternatively, using all tests as the CFF, Algorithm 1 determines that the only sick students are 1 and 4 (using Proposition 3.3 or by inspection).

#### Example 2

**Outbreak in high school - realistic example** Consider the setup of Example 1 in a more general scenario where courses are divided into *P* time periods, each time period has *C* courses with *S* students per course, and let $\mathcal {E}$ be the corresponding edges of the hypergraph. Then, since each time period forms a colour class (i.e. $\ell =P$), an $(\mathcal {E},1$)-CFF(*t*, *CS*) can be built with $t=P\times (t(1,C) +S) = O(P\times (S+\log C))$. In particular, let us take a realistic scenario of a high school with students taking 4 courses each term, like the ones in Ontario, Canada. Suppose $n=900$ students take $P=4$ courses each, each course having 30 students for a total of $m=120$ courses. We vertically concatenate 2*P* matrices. For time period *i*, we use $M_i$ (built by repeating columns of a 1-CFF$(7,30=120/4)$) and $N_i$ (formed by identities of order 30 side-by-side, pasted under the edges in *i*). Assume there is an outbreak in a single course, involving any number of students ($\le 30$) in that course. We only need $7\times 4 = 28$ tests to determine the course where the outbreak took place ($M_i$ build from 1-CFF(7,30), $1\le i \le 4$). A total of $28 + 30\times 4 = 148$ tests can be used to identify all infected individuals (up to 30) in this set of 900 students. Note that our assumption is that there is *r*=1 course that contains all infected individuals, even though there may be many infected courses (say up to 90 other courses that the infected students also take in other time periods). In other words, the hypergraph is assumed to have a defective cover of size $r=1$, but it is possible that up to 91 edges are defective.

### Hypergraphs with overlapping edges and defect cover of general size

For $r>1$, we need to use strong edge-colourings to be able to split the problem according to colour classes without too many infected edges appearing in the same colour class. The next theorem and corollary generalize analogous results in Sect. 5.1 to the case of $r>1$.

#### Theorem 5.3

(overlapping edges, general *r*)  Let $\mathcal {H}=([1,n],\mathcal {E})$ be a hypergraph and let $r\ge 2$ be an upper bound on the number of edges of a minimum defective cover. Let $\mathcal {C}_1, \mathcal {C}_2, \ldots , \mathcal {C}_{s'}$ be the sets of edges in each colour class of an $s'$-strong-edge-colouring of $\mathcal {H}$. Let $k_i=\max \{|S|:S\in \mathcal {C}_i\}$. Then there exists an $(\mathcal {E},r)$-CFF(*t*, *n*) with $t = \sum _{i=1}^{s'} (t(r,|\mathcal {C}_i|) + k_i t(r-1,|\mathcal {C}_i|)).$

#### Proof

The construction is similar to the one in Theorem 5.1 in that we deal with each colour class separately. Let $t_i=t(r,|\mathcal {C}_i|)$ and $t_i'=t(r-1,|\mathcal {C}_i|)$. For each colour class $\mathcal {C}_i \subseteq \mathcal {E}$, $1\le i \le s'$, we build two matrices $M'_i$ and $N'_i$, following the construction of Theorem 4.4, with $t_i$ rows and $t_i' k_i$ rows, respectively. For any set of edges $E=\{e_1, \ldots , e_r\}\subseteq \mathcal {E}$, there are at most *r* edges in $\mathcal {C}_i$ which intersect any edge in *E*, due to the definition of strong colouring.

Let $V_i=\cup _{e \in \mathcal {C}_i} e$ and $X_i=[1,n]\setminus V_i$, for each $1\le i \le s'$. Let $\mathcal {H'}_i=(V_i,\mathcal {C}_i)$. Since $\mathcal {H'}_i$ is a hypergraph with non-overlapping edges, the construction in the proof of Theorem 4.4 gives a $( \mathcal {C}_i,r)$-ECFF$(t_i,|V_i|)$ $M'_i$ and a $(\mathcal {C}_i,r)$-InCFF$(t_i' k_i,|V_i|)$ $N'_i$. Let $\mathcal {H}_i=([1,n],\mathcal {C}_i)$, which has the same edges of $\mathcal {H}'_i$ without removing the isolated vertices in $X_i$. Extend $M'_i$ to matrix $M_i$ by adding one column of 0s for each vertex in $X_i$; similarly, extend $N'_i$ to matrix $N_i$ by adding one column of 0s for each vertex in $X_i$. The vertical concatenation of $M_i$ and $N_i$ is enough to determine the status of each item contained in any of the edges in colour class $\mathcal{C}_i$.

Vertically concatenate $M_1, M_2, \ldots , M_{s'}, N_1, N_2, \ldots , N_{s'}$. We must show that this is an $(\mathcal {E},r)$-CFF($\sum _{i=1}^{s'}(t_i+ t'_ik_i),n$). Let $I\subseteq \cup _{j=1}^{\ell } e_j$, $\ell \le r$ and let $i_0\in [1,n]\setminus I$.

If there exists an edge *e* such that $i_0 \in e$ and $e\cap I=\emptyset$, we claim that in $M_i$ corresponding to the colour class $\mathcal {C}_i\supset \{e\}$, we can find a row *x* where $M_i[x,i_0]=1$ and $M_i[x,y]=0$ for all $y\in I$. This is because in this colour class there are at most $\ell \le r$ edges that intersect *I* (due to strong colouring and the fact that $I\subseteq \cup _{j=1}^{\ell } e_j$), and since $M_i$ was built from an *r*-CFF on the edges of this colour class, in some row *x* the columns corresponding to edge *e* have 1’s, while the columns corresponding to vertices in the other edges intersecting *I* must have zeros; in addition, vertices $v\in I\setminus \cup _{e\in \mathcal{C}_i} e$ have 0 in row *x* by construction.

The other case to analyze is when $i_0\in e$ such that $e\cap I\not =\emptyset$. Let $\mathcal {C}_i$ be the same colour class that edge *e* belongs to. In $N_i$ we claim that we can find a row *x* where $N_i[x,i_0]=1$ and $N_i[x,y]=0$ for all $y \in I$. Let $I_1=I\cap V_i$ and $I_2=I\setminus V_i$, so that $I=I_1\cup I_2$ is partitioned into vertices that occur in colour class $\mathcal {C}_i$ and vertices that don’t. Due to strong colouring, there are at most $\ell \le r$ edges in $\mathcal {C}_i$ that intersect any of $e_1, \ldots , e_{\ell }$. Therefore, there are at most $\ell \le r$ edges in $\mathcal {C}_i$ that intersect $I_1$, one of which is *e*; let us call these edges $e,e_1', e_2', \ldots ,e_{\ell -1}'$. Since $N'_i$ is an $(\mathcal {C}_i,r)$-InCFF, there is a row *x* of $N_i$ such that $N_i[x,i_0]=1$ and $N_i[x,y]=0$ for all $y\in I_1$. In addition, by construction $N_i[x,y]=0$ for all $y\in I_2$. Thus, we proved the claim. This concludes the proof. $\square$

**Fig. 4:**
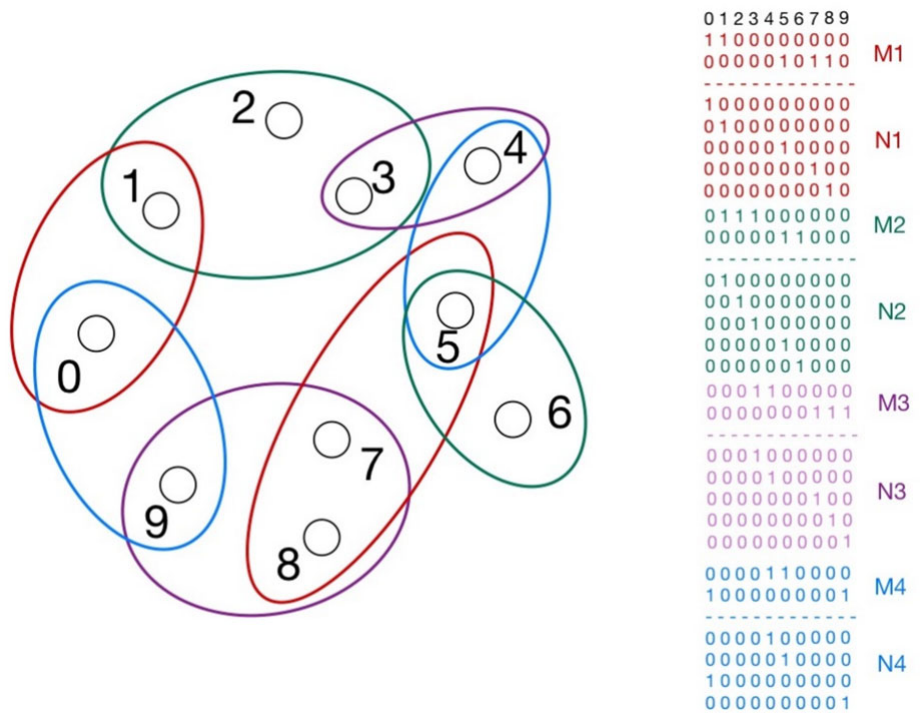
Example of an application of Theorem 5.3 for a graph with a strong edge colouring with the following colour classes: $C_1=\{\{0,1\},\{5,7,8\}\}, C_2=\{\{1,2,3\},\{5,6\}\}, C_3=\{\{3,4\},\{7,8,9\}\}, C_4=\{\{0,9\},\{4,5\}\}$. Due to the small size of colour classes, the 1-CFF and 2-CFF used as ingredients are identity matrices $I_2$

An example of an application of Theorem 5.3 is shown in Figure 4.

#### Corollary 5.4

Let $\mathcal {H}=([1,n],\mathcal {E})$ be a *k*-uniform hypergraph. Then, we can build an $(\mathcal {E},r)$-CFF(*t*, *n*) with $t(r,\mathcal {E}) \le \chi '_s(\mathcal {H})\times (t(r,\lfloor n/k\rfloor )+k t(r-1,\lfloor n/k\rfloor ))$.

#### Proof

We can apply Theorem 5.3 with $s'=\chi '_s(\mathcal {H})$, $k_i=k$ for all $1\le i\le \ell$ and note that each colour class contains at most $\lfloor n/k \rfloor$ edges so that $|\mathcal {C}_i| \le \lfloor n/k \rfloor$. $\square$

#### Example 3

Consider the scenario of Example 1 and $r=2$. We can find a strong colouring for the hypergraph of that example with $s'=6$ colours with colour classes: $\{course 1, course 4\}, \{course 2, course 5\}, \{course 3,course 6\},\{ course 7, course 10\},$ $\{course 8,course 11 \}, \{course 9,course 12\}.$ For each colour class, we can use identity matrices $I_3$ as the 2-CFF(3, 3) and 1-CFF(3, 3) required. If there are outbreaks in 2 courses, any set of up to 6 infected students in these 2 courses can be detected with 36 tests. This is a toy example, and of course, 36 tests are more tests than testing the 18 students individually. If we keep the same edge configuration, but substitute each student by 6 students, then each edge has 18 students. The 1-CFF(3, 3) can be substituted by an 1-CFF(6, 15). In this case, 54 tests are sufficient to detect up to 36 infected students from an outbreak in 2 classes among these 108 students.

Next, we discuss a simple construction using strong vertex colouring. For some types of hypergraphs, Theorem 5.5 gives better bounds than the results based on strong edge colouring. See Sect. 5.3 for some applications of this theorem.

#### Theorem 5.5

Let $\mathcal {H}=([1,vs],\mathcal {E})$ be a hypergraph that has an *s*-strong-vertex-colouring such that each colour class has cardinality *v*. Suppose the vertices in [1, *vs*] are labeled (*c*, *x*) where $1 \le c \le s$ and $1\le x \le v$, and corresponding to columns in the order $(1,1), \ldots , (1,v), (2,1),\ldots , (2,v), \ldots , (s,1), \ldots , (s,v)$. Let $r\ge 1$. If *A* is an *r*-CFF(*t*, *v*), then $B=I_s\otimes A$ is an $(\mathcal {E},r)$-CFF(*st*, *sv*), which gives the upper bound $t(r,\mathcal {E}) \le s \times t(r, v)$.

#### Proof

Matrix *B* is of the form

$$B=\left[ \begin{array}{ccccc} A & \textbf{0} & \textbf{0}& \cdots & \textbf{0}\\ \textbf{0} & A & \textbf{0} & \cdots & \textbf{0} \\ \vdots & \vdots & \ddots & \vdots & \vdots \\ \textbf{0} & \textbf{0} & \cdots & A & \textbf{0} \\ \textbf{0} & \textbf{0} & \cdots & \textbf{0} & A \end{array}\right] $$

with rows labelled as (*c*, *x*), for $1\le c\le s$ and $1\le x\le t$ and columns ordered as specified, where $\textbf{0}$ is a matrix of all zeros of the appropriate dimension. Let $\{e_1,\ldots ,e_r\} \subseteq {\mathcal {E}}$, $E=\cup _{i=1}^r e_i$, $I\subseteq E$, and $i_0\in [1,n]\setminus I$. Let $y=|I|.$ We need to show that there exists a row *z* of *B* where $B_{z,i_0}=1$ and $B_{z,j}=0$ for all $j\in I$. We first consider the case $i_0=(c_0,x_0)\not \in E$. Because *A* is an *r*-CFF and *I* contains at most *r* vertices of colour $c_0$, there exists a row *p* such that $A_{p,x_0}=1$ and $A_{p,x}=0$, for all *x* such that $(c_0,x)\in I$. Thus in *B*, the row *p* of row block $c_0$ (i.e. row $z=(c_0,p)$ of B) will have $B_{(c_0,p),(c_0,x_0)}=1$ and $B_{(c_0,p),(c_0,x)}=0$ for all $(c_0,x)\in I$. In addition, by construction, $B_{(c_0,p),(c,x)}=0$ for all $(c,x) \in I$, $c \not =c_0$. We now consider the case $i_0=(c_0,x_0) \in E\setminus I$; thus $i_0 \in e_i$, for some $1\le i \le r$. We know there exists a row $1\le p \le t$ such that $A_{p,x_0}=1$ and $A_{p,x}=0$, for all $(c_0,x)\in I$, since there are at most $r-1$ such *x* and *A* is an *r*-CFF. Thus in *B*, the row *p* at row block $c_0$ is such that $B_{(c_0,p),(c_0,x_0)}=1$ and $B_{(c_0,p),(c_0,x)}=0$ for all $(c_0,x)\in I$. In addition, by construction, $B_{(c_0,p),(c,x)}=0$ for all $(c,x) \in I$, $c \not =c_0$. This completes the proof. $\square$

Theorem 5.5 can be easily extended to the case of colour classes of different sizes; the proof argument is the same as in Theorem 5.5. This result is presented next.

#### Theorem 5.6

Let $r\ge 1$. Let $\mathcal {H}=([1,n],\mathcal {E})$ be a hypergraph with an *s*-strong-vertex-colouring such that each colour class has cardinality $v_c$, $1\le c \le s$. Suppose the vertices in [1, *n*] are labeled (*c*, *x*) where $1 \le c \le s$ and $1\le x \le v_c$, and corresponding to columns in the order $(1,1), \ldots , (1,v_1), \ldots , (s,1), \ldots , (s,v_s)$. Let $A_c$ be an *r*-CFF$(t_c,v_c)$, and let $t=\sum _{c=1}^s t_c$. Then,

$$B=\left[ \begin{array}{ccccc} A_1 & \textbf{0} & \textbf{0}& \cdots & \textbf{0}\\ \textbf{0} & A_2 & \textbf{0} & \cdots & \textbf{0} \\ \vdots & \vdots & \ddots & \vdots & \vdots \\ \textbf{0} & \textbf{0} & \cdots & A_{s-1} & \textbf{0} \\ \textbf{0} & \textbf{0} & \cdots & \textbf{0} & A_s \end{array}\right] $$

is an $(\mathcal {E},r)$-CFF(*t*, *n*).

### Cover-free families for sets of consecutive defectives on hypercubes

In this section, we construct CFFs for the hypergraph where edges are “nearby” vertices in a hypercube. This is an application of Theorem 5.5.

**Fig. 5:**
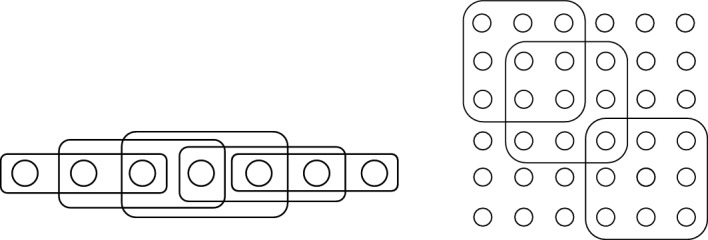
Example of CH(1, 7, 3) with a total of 7 vertices and 5 edges on the left; and CH(2, 6, 3) with a total of 36 vertices and 16 edges on the right (only 3 edges displayed)

Consider a hypergraph where defective items appear close together when seen as points in a hypercube of dimension $\delta$ and side *L*. The edges correspond to sub-hypercubes of the same dimension and side $\ell \le L$. For one-dimensional hypercubes of side *L*, this is related to burst errors, i.e. up to $\ell$ errors occurring consecutively among *L* points on a line; for $\delta =2$, the edges are $\ell \times \ell$ squares on an $L\times L$ grid. Figure 5 shows examples of consecutive hypergraphs.

Let the *consecutive hypercube hypergraph* of dimension $\delta$, side *L* and edge side $\ell \le L$, denoted $CH(\delta , L, \ell )$, be the hypegraph with vertex set $V_{\delta ,L}=[1,L]^\delta$, edges of the form $e_x=\{y \in V_{\delta ,L}:0\le y_i-x_i<\ell , \mathrm {for\ all\ } 1\le i \le \delta \}$ and edge set $\mathcal {E}_{\delta , L, \ell }=\{ e_x : x\in V_{\delta ,L} \}$. Note that $e_x$ corresponds to the $\ delta$-dimensional hypercube of side $\ell$ where *x* is the point where each coordinate is the smallest among points in this edge and $|e_x|=\ell ^\delta$.

An $(\mathcal {E}_{\delta , L, \ell },r)$-CFF aims at locating defective items spread across *r* $\delta$-dimensional hypercubes of side $\ell$ over a $\delta$-dimensional hypercube of side *L*.

#### Theorem 5.7

(consecutive hypercube hypergraph) Let $r\ge 1$. For integers $\delta \ge 1$, $\ell \ge 2$, $L > \ell$, let $\mathcal{H}=([1,L^{\delta }],\mathcal {E}_{\delta , L, \ell })$ be a $CH(\delta , L, \ell )$. Then

$$\begin{aligned} t(r, \mathcal {E}_{\delta , L, \ell }) \le \ell ^\delta \times t(r, \lceil v \rceil ^{\delta }),\ \ \textrm{where}\ v = \frac{L}{\ell }. \end{aligned}$$

#### Proof

This hypergraph has strong vertex-chromatic number $\chi (CH(\delta , L, \ell ))=\ell ^{\delta }$, since $\ell ^{\delta }$ is the edge size and $\ell ^{\delta }$ colours are enough to colour this graph: for vertex $(x_1, x_2,\ldots ,x_{\delta })$ assign colour $(x_1\mod \ell , x_2\mod \ell , \ldots , x_{\delta }\mod \ell )$. Each colour class has at most $\lceil \frac{L}{\ell } \rceil ^{\delta }$ vertices. The result follows from applying Theorem 5.5, yielding a $(\mathcal {E}_{\delta , L, \ell },r)$-CFF$(t, L^\delta )$ with $t = \ell ^\delta \times t(r,\lceil \frac{L}{\ell } \rceil ^{\delta })$. $\square$

#### Example 4

**Fig. 6:**
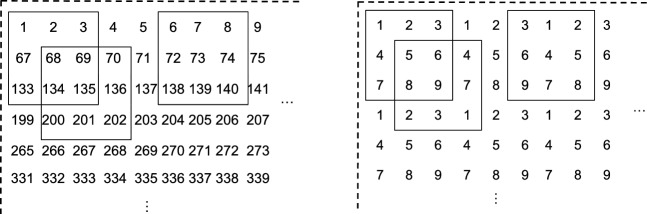
Hypergraph CH(2, 66, 3) has 4356 vertices and edges consisting of every $3\times 3$ subsquare. Each vertex in the left picture is assigned the corresponding colour displayed in the right picture, so the chromatic number is 9

Consider a venue with 4356 people sitting in a square auditorium of 66 rows with 66 seats per row. Edges are sets of individuals sitting nearby. We consider edges of size 9 consisting of all possible contiguous $3 \times 3$ squares (see Fig. 6). There are at most 9 edges passing through each vertex corresponding to the $3\times 3$ subsquares, only some edges are shown in Fig. 6. This example corresponds to $\mathcal {H}=CH(2, 66, 3)$ with chromatic number $\chi (H)=9$, and $v=22$. Let us take a 2-CFF(40, 512) obtained from Proposition 6.2, using $q=8$ and $k = 2$; drop columns arbitrarily to form *A* a 2-CFF$(40,22^2=484)$. By Theorem 5.5 and Theorem 5.7, $I_9 \otimes A$ is an $(\mathcal {E},2)$-CFF(360, 4356). Therefore, 360 tests are enough to screen 4356 people, where at most two regions of size $3\times 3$ contains all infected individuals.

For the case of $\delta =1$, we consider a cyclic generalization of the consecutive hypercube hypergraph $CH(1, L, \ell )$. Let *Cycl*-*H* be the hypergraph $(V=[0,L-1],\mathcal {E}_{cycl})$ where $\mathcal {E}_{cycl}=\{ \{i,i+1,\ldots , (i+(\ell -1))\}_{\textrm{mod}\ L}: i \in V\}$.

#### Corollary 5.8

Let *Cycl*-$H=(V=[0,L-1],\mathcal {E}_{cycl})$, and $e=L\mod \ell$. Then, there exists an $(\mathcal {E}_{cycl},r)$-CFF(*t*, *L*), where $t=(\ell +e) \cdot t(r, \lceil L/\ell \rceil )$.

#### Proof

Since there is a strong vertex colouring with $\ell +e$ colours, the result follows an analogous proof to the one of Theorem 5.7. $\square$

## Swiss-Army-Knife CFFs

In this section, we focus on constructions for CFFs on hypergraphs that serve several purposes, and call them *Swiss-army-knife constructions*. For example, a matrix *M* can be both an $(\mathcal{E},r^-)$-CFF and an $(\mathcal {E},r^+)$-ECFF for $r^+>r^-$ for a hypergraph; in addition, *M* can be a plain *d*-CFF. More concretely, let us consider the case of disease screening in a school with 30 disjoint classrooms of size 30. If *M* is simultaneously a $(\mathcal {E},2)$-CFF, a $(\mathcal {E},30)$-ECFF, and a 4-CFF, we can obtain less or more information depending on which assumptions are satisfied. If the set of infected individuals is contained in 2 classrooms, we can identify up to 60 such infected individuals. On the other hand, if only 4 individuals are infected but belong to 4 different classrooms, then the infected individuals can also be determined. Otherwise, the set of infected classes can always be determined. In this section, we discuss constructions of Swiss-army-knife CFFs.

We start with a simple array construction to showcase this type of CFFs. An array-based scheme for group testing uses an $n_1 \times n_2$ array, where each entry of the array corresponds to an item to be tested and the tests are performed on rows and columns, for a total of $n_1+n_2$ tests. This can be used on a 2-stage algorithm, where all items at the intersection of a positive row and column should be individually tested in a second stage to solve ambiguities (Hudgens and Kim 2011; Kim et al. 2007; Phatarfod and Sudbury 1994). For $d=1$, one stage is enough.

**Fig. 7:**
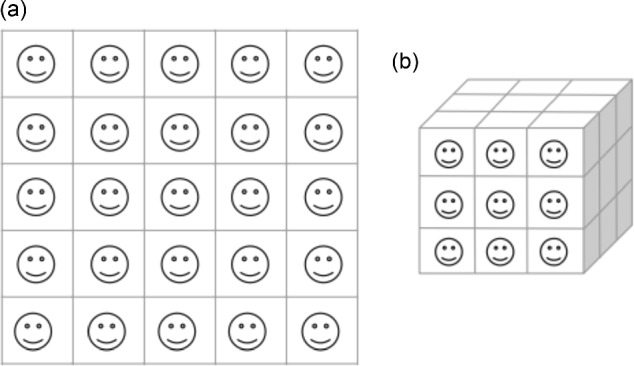
(a) A $5\times 5$ array GT with 25 items and 10 tests. (b) A $3\times 3\times 3$ hypercube GT with 27 items and 9 tests

Figure 7 (a) shows a $5\times 5$ array that can be seen as a 1-CFF(10, 25), which allows us to identify one infected element with 10 tests. This can also be considered an $(\mathcal {E},1)$-CFF(10, 25) where the set of edges $\mathcal {E}$ corresponds to the rows (or columns) of the array. In this case, we can identify up to 5 defective items, as long as they all belong to the same edge. Moreover, this can also be considered an $(\mathcal {E},5)$-ECFF(10, 25), where we can identify up to 5 infected edges, but we cannot perform internal identification.

**Fig. 8:**
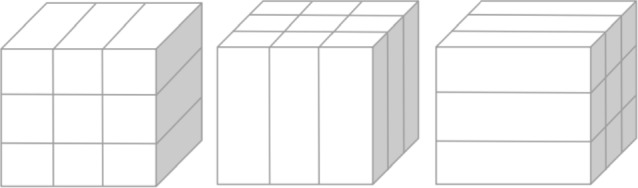
The 27 hyperedges of a $3\times 3\times 3$ hypercube, each with 3 elements

**Fig. 9:**
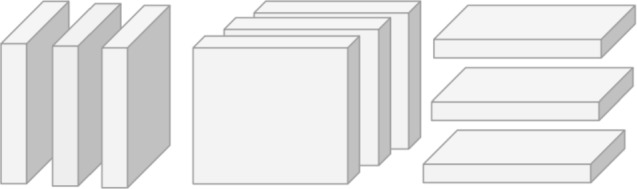
The 9 tests of a $3\times 3\times 3$ hypercube, each test with 9 elements, each element belonging to 3 tests

This idea can be generalized to higher dimensions, constructing an $n_1\times \ldots \times n_k$ hypercube (Berger et al. 2004; Kim and Hudgens 2009), which is a 1-CFF$(n_1+\ldots +n_k,n_1\times \ldots \times n_k)$. Figure 7 (b) shows a 3-dimensional hypercube, where each point represents an item and tests are given by fixing the value of one dimension as shown in Figure 9. If all defective items are clustered in either a row or a column in a 2-dimensional sub-array, we can precisely identify all of them in one round; thus, this is a vertex-identifying $(\mathcal {E},1)$-CFF$(3n,n^3)$ where the set of edges corresponds to one dimensional sub-arrays, as shown in Figure 8.

We generalize this for higher dimensions in the next proposition. To simplify the notation, we take $n_1=\ldots =n_k=n$, but the next results are valid for the general case. An $[n]^k$-hypercube group testing matrix is an 1-CFF$(kn,n^k)$ matrix defined as follows. Items are in $\mathbb {Z}_n^k$ and rows/tests are given by $T_{v,a}=\{x\in \mathbb {Z}_n^k: x_v=a \}$, $1\le v \le k$, $a\in \mathbb {Z}_n$. Denote $x(v)=(x_1,\ldots ,x_{v-1},x_{v+1},\ldots ,x_k)$ for $x\in \mathbb {Z}_n^k$, $1\le v\le k$.

### Proposition 6.1

Let *A* be an $[n]^k$-hypercube group testing matrix. Let $\mathcal {H}_v=([1,n]^k,\mathcal {E}_v)$ where $\mathcal {E}_v=\{\{x\in \mathbb {Z}_n^k: x(v)=(a_1,\ldots ,a_{k-1})\}: (a_1,\cdots , a_{k-1})\in \mathbb {Z}_n^{k-1}\}$, $1\le v \le k$, and let $\mathcal {H}=([1,n]^k,\mathcal {E})$ where $\mathcal {E}=\mathcal {E}_1 \cup \cdots \cup \mathcal {E}_k$. Then, for any $1\le v \le k$, *A* is an $(\mathcal {E}_v,1)$-CFF($kn,n^k$) and if $k=2$, *A* is also an $(\mathcal {E}_v,|\mathcal {E}_v|=n)$-ECFF($2n,n^2$). Moreover, *A* is an $(\mathcal {E},1)$-CFF($kn,n^k$).

### Proof

For ECFF, note that when $k=2$ each edge is tested in a different test containing only the vertices of the edge, therefore we have an $(\mathcal {E}_v,|\mathcal {E}_v|=n)$-ECFF($2n,n^2$). For vertex-identifying CFFs, if defectives are contained in an edge of $E_v$, vertices in all other edges of $E_v$ will be in passing tests, and tests $T_{v,a}$, $a \in \mathbb {Z}_n$, reveal the defective status of each item in $E_v$, which gives an $(\mathcal {E}_v,1)$-CFF($kn,n^k$). Because *A* is an $(\mathcal {E}_v,1)$-CFF($kn,n^k$), for every $1\le v\le k$, then *A* is an an $(\mathcal {E},1)$-CFF($kn,n^k$). $\square$

### Construction from polynomials

We now consider a construction of CFFs given independently by Kautz and Singleton (1964) and Erdös et al. (1985), which uses polynomials over finite fields and is related to Reed-Solomon codes. Let *q* be a prime power, *k* a positive integer, and $\mathbb {F}_q = \{a_1, \ldots , a_q\}$ be a finite field. Then, for $d \le \frac{q-1}{k}$, a *d*-CFF($t=q^2, n=q^{k+1}$) $\mathcal {F}$=($X, \mathcal {B}$) is defined as follows: $X = \mathbb {F}_q \times \mathbb {F}_q,$ and, letting $B_f = \{(a_1, f(a_1)), \ldots , (a_q, f(a_q))\},$

7 $$\begin{aligned} \mathcal {B} = \{B_f: f \in \mathbb {F}_q[x]_{\le k}\}. \end{aligned}$$

We restrict the CFF matrix to *i* *blocks* of rows by taking $X = \{a_1, \ldots , a_i\} \times \mathbb {F}_q$, $B_f(i) = \{(a_1, f(a_1)), \ldots , (a_i, f(a_i))\}$ and $\mathcal {B}(i) = \{B_f(i): f \in \mathbb {F}_q[x]_{\le k}\}$, which yields the following result.

#### Proposition 6.2

(Idalino and Moura (2019); Wei (2006)) Let *q* be a prime power, $k\ge 1$ and $q \ge dk+1$, and let *M* be the *d*-CFF($q^2, q^{k+1}$) obtained in Eq.7. If we restrict *M* to the first $(d'k+1)$ blocks of rows, we obtain a $d'$-CFF($(d'k+1)q, q^{k+1}$), for any $d'\le d$.

For instance, for $q=5$ and $k=1$, if we restrict a 4-CFF($5^2,5^2$) to its first 2 blocks of rows, we get a 1-CFF($2\times 5, 5^2$), with 3 blocks of rows we get a 2-CFF($3\times 5, 5^2$), etc. Next, we show that this construction is a vertex-identifying CFF, for an appropriate hypergraph, that can tolerate as many as *q* defective items (as long as inside an edge) with as few as $(k+1)q$ tests. When $k=1$, the same construction is also an edge-identifying CFF that can tolerate as many as *q* defective edges with as few as 2*q* tests.

#### Theorem 6.3

Let $k\ge 1$ and *q* be a prime power such that $q\ge k+1$. Let $\mathcal {E}= \{e_1,\ldots ,e_{q^k}\}$ be a set-partition of [1, *n*] such that $|e_i|=q$ for all $1 \le i \le q^k$. Then,

1. there exists an $(\mathcal {E},1)$-CFF$((k+1)q, q^{(k+1)})$;
2. if $k=1$, there exists a 1-CFF$(2q, q^{2})$, which is also an $(\mathcal {E},q)$-ECFF$(2q, q^{2})$.

Moreover, if $q \ge dk+1$ where $d \ge 1$, then

3. there exists a *d*-CFF$((dk+1)q, q^{(k+1)})$ which is also a $(\mathcal {E},1)$-CFF$((dk+1)q, q^{(k+1)})$;
4. if $k=1$, there exists a *d*-CFF$((d+1)q, q^{2})$ which is also a $(\mathcal {E},q)$-ECFF$((d+1)q, q^{2})$.

#### Proof

Let *M* be the incidence matrix of the set system in Eq. 7, where $M_{(x,y),p}=1$ if and only if $p(x)=y$. Letting $\mathbb {F}_q=\{a_1, \ldots , a_q\}$, we identify each edge in $\mathcal {E}$ with a set $S_{i_1,\ldots ,i_k}=\{ p \in \mathbb {F}_q[x] : p(a_j) = a_{i_j}, for\ 1\le j \le k \}$, for $(i_1,\ldots ,i_k)\in [1,q]^k$. In other words, each edge corresponds to a set of *q* polynomials that have the same evaluation for elements $a_{1}, \ldots , a_{k}$.

Now we prove that this is an $(\mathcal {E},1)$-CFF and we only need $(k+1)q$ tests to identify all the errors inside a single edge. Let $I \subseteq e_i$ for any $e_i \in \mathcal {E}$. We need to show that for any column $p \notin I$, there exists a row (*c*, *d*) s.t. $p(c) = d$ and $f(c) \ne d$, for all $f \in I$. There are two cases to be considered:

Case i) $p \in e_i \setminus I$: Take $(c,d)=(a_{k+1},p(a_{k+1}))$. We know for any $f\in I$, $f(a_{k+1})\ne p(a_{k+1})$; for otherwise, since they already have the same evaluation for $a_{1}, \ldots , a_{k}$, this would imply they would be the same polynomial.

Case ii) $p \not \in e_i$: Let $\ell$ be the smallest index such that $f(a_\ell ) \ne p(a_{\ell })$. We know that, by the edge definition, $\ell \le k$, otherwise *f* and *p* would be on the same edge (this is Case i)). Therefore, take $(c,d)=(a_{\ell },p(a_{\ell }))$. This concludes the proof that this is an $(\mathcal {E},1)$-CFF($(k+1)q, q^{(k+1)}$), which is part 6.3.

If $k=1$, the first block of rows in *M* has each test coinciding with each edge. Therefore, *M* is also a $(\mathcal {E},q)$-ECFF($2q, q^2$), which is also a 1-CFF($2q, q^2$) by Proposition 6.2. This concludes the proof of part 6.3.

In the case of $q\ge dk+1$ where $d\ge 1$, by Proposition 6.2, we have a *d*-CFF($(dk+1)q, q^{(k+1)}$), and by part 6.3 the first $k+1$ blocks of rows of this matrix yields a $(\mathcal {E},1)$-CFF($(k+1)q, q^{(k+1)}$), and so thus the full matrix. This concludes part 6.3. Similarly, in the case $k=1$, Proposition 6.2 and part 6.3 yield part 6.3. $\square$

#### Example 5

Let $q=5$ and $k=1$. Then, we have a 1-CFF($2q = 10, q^{2} = 25$). Moreover, let $\mathcal {E} = \{e_1, e_2, e_3, e_4, e_5\}$, where $e_{1} = \{0, x, 2x, 3x, 4x\}, e_{2} = \{1, x+1, 2x+1, 3x+1, 4x+1\}, e_{3} = \{2, x+2, 2x+2, 3x+2, 4x+2\}, e_{4} = \{3, x+3, 2x+3, 3x+3, 4x+3\}, \text { and } e_{5} = \{4, x+4, 2x+4, 3x+4, 4x+4\}$. This gives us an $(\mathcal {E},1)$-CFF($2q = 10, q^{2} = 25$) that is also an $(\mathcal {E},5)$-ECFF(10, 25).

#### Example 6

Let $q=3$ and $k=2$. Then, we have a 1-CFF($3q = 9, q^{3} = 27$). Moreover, let $\mathcal {E} = \{e_1, e_2, \ldots , e_9\}$, where $e_{1} = S_{1,1} = \{0, x^2+2x, 2x^2+x\}, e_{2} = S_{1,2} = \{x, x^2, 2x^2+2x\}, \ldots , e_{9} = S_{3,3}= \{2, x^2+2x+2, 2x^2+x+2\}$. Note that all polynomials *p* in $S_{i, j}$ have $p(a_1) = a_i$ and $p(a_2) = a_j$, for all $1 \le i, j \le 3$. This gives us an $(\mathcal {E},1)$-CFF($3q = 9, q^{3} = 27$).

#### Example 7

Let $q=5$, $k=2$, and $d = 2$. We have a 2-CFF($q^2 = 25, q^3 = 125$) that is also a $(\mathcal {E},1)$-CFF($q^2 = 25, q^{3} = 125$), where $\mathcal {E} = \{e_1=S_{1,1}, e_2=S_{1,2}, \ldots , e_{25}=S_{5,5}\}$, with $|e_i| = 5$, $1\le i \le 25$.

Note that the construction in Theorem 6.3 is equivalent to a $[q]^{k+1}$-hypercube, but it is more flexible since we can add more tests (Proposition 6.2) for a total of $(dk+1)q$ tests, where $q\ge dk+1$, to obtain both an $(\mathcal {E},1)$-CFF$((dk+1)q,q^{k+1})$ and a *d*-CFF$((dk+1)q,q^{k+1})$, so any *d* defects anywhere or $q>d$ defects inside an edge can be found.

### Constructions from designs

A 2-(*v*, *k*, 1) packing design is a set of *k*-subsets called blocks of a *v*-set of points such that no two points appear together in more than one block. A packing design with $k\ge 3$ and *b* blocks yields a 2-CFF(*v*, *b*). In particular, 2-(*v*, *k*, 1) designs (where every two points appear in exactly one block) yield a 2-CFF$(v, {v \atopwithdelims ()2}/{k\atopwithdelims ()2})$ (Idalino and Moura 2024; Wei 2006). Such a design is *resolvable* if its blocks can be partitioned into resolution classes, which are sets of disjoint blocks whose union is the *v*-set.

#### Proposition 6.4

If there exists a resolvable 2-(*v*, *k*, 1) design (which has $b={v \atopwithdelims ()2}/{k\atopwithdelims ()2}$ blocks and $\frac{v-1}{k-1}$ blocks passing by each point) then there exists a matrix *M* that is an $(\mathcal {E},1)$-CFF$(v+\frac{v-1}{k-1},{v \atopwithdelims ()2}/{k\atopwithdelims ()2})$, for $\mathcal {E}$ consisting of $\frac{v-1}{k-1}$ non-overlapping edges of size $d = v/k$, that is also an $(\mathcal {E},|\mathcal {E}|)$-ECFF$(v+\frac{v-1}{k-1},{v \atopwithdelims ()2}/{k\atopwithdelims ()2})$ and a 2-CFF$(v+\frac{v-1}{k-1},{v \atopwithdelims ()2}/{k\atopwithdelims ()2})$. In particular, for every $v\equiv 3\ (\textrm{mod}\ 6)$, there exists a matrix *M* that is a $(\mathcal {E},1)$-CFF$(\frac{3v-1}{2},v(v-1)/6)$ for $\mathcal {E}$ consisting of $(v-1)/2$ edges of size *v*/3, which is also an $(\mathcal {E},|\mathcal {E}|)$-ECFF$(\frac{3v-1}{2},v(v-1)/6)$ and a 2-CFF$(\frac{3v-1}{2},v(v-1)/6)$.

#### Proof

The hypergraph considered has $b={v \atopwithdelims ()2}/{k \atopwithdelims ()2}$ vertices in correspondence with each block of the design and $\frac{v-1}{k-1}$ disjoint edges of cardinality *v*/*k* in correspondence with each resolution class of the design. Create *M* by vertically concatenating the incidence matrix of the 2-(*v*, *k*, 1) resolvable BIBD with one row/test for each resolution class that tests all blocks of that class. It is already known that the first part of the matrix is a 2-CFF$(v,{v \atopwithdelims ()2}/{k\atopwithdelims ()2})$ (Idalino and Moura 2024; Wei 2006). The tests for each individual resolution class can totally identify the defective edges, noting that Algorithm 2 returns a boolean *z*. Thus, *M* is an $(\mathcal {E},|\mathcal {E}|)$-ECFF$(v+\frac{v-1}{k-1},{v \atopwithdelims ()2}/{k\atopwithdelims ()2})$. If only one edge is defective, the status of tests for each of the points $1, 2, \ldots , v$ (first *v* rows) is enough to determine which vertices in the edge are defective. This is true because for any block *B* (any vertex) and any point $x\in B$, the test corresponding to *x* includes vertex *B*, but does not include any other vertex that is in the same resolution class as *B*. Thus, *M* is also an $(\mathcal {E},1)$-CFF$(v+\frac{v-1}{k-1},{v \atopwithdelims ()2}/{k\atopwithdelims ()2})$. In particular, since a resolvable Steiner triple system exists for every $v\equiv 3 \ (\textrm{mod}\ 6)$, this is precisely a resolvable 2-(*v*, 3, 1) design, which completes the proof. $\square$

**Fig. 10:**
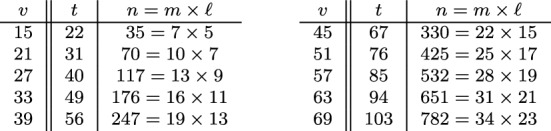
Swiss-army-knife CFF parameters in Proposition 6.4 using resolvable STS(*v*)

In Figure 2, matrix *A* is the incidence matrix of a resolvable 2-(9, 3, 1) design, which has $b=12$ blocks, 4 resolution classes and 3 blocks per resolution class. This can be used to test a hypergraph with 4 non-overlapping edges with 3 vertices each. Matrix *M* given by Proposition 6.4 has $t=13$ rows and $n=b=12$ columns, which is not interesting since the identity matrix (individual tests) has fewer rows and is more powerful. On the other hand, more interesting examples of this construction using resolvable 2-(*v*, 3, 1) designs (which are resolvable Steiner triple systems of order *v*, STS(*v*)) for a larger number of points *v* are shown in Figure 10. Using these examples, *m* disjoint groups (edges), with $\ell$ individuals (vertices) each, can be screened with *t* tests. The corresponding matrices have *t* rows and *n* columns, and are simulataneously a 2-CFF, an $(\mathcal {E},|\mathcal {E}|=m$)-ECFF and an $(\mathcal {E},1)$-CFF. For instance, as displayed in Figure 10, we can use 103 tests to screen for a contagious disease in a school with 782 students that are divided into 34 classrooms with 13 students each. The test results will reveal precisely which classes contain one or more infected students. In addition, if this infection is contained in a single classroom, it will be possible to know which ones of the 13 students are sick; otherwise, if only two individuals are sick across the whole school they can also be identified.

*Remark about Swiss-army-knife CFFs. *One may wonder whether these constructions of Swiss-army-knife CFFs are too specific and have limited applicability. It may be so, but the purpose of this section is to illustrate how awareness of the structure of the group testing problem can help in the design of an experiment that yields more information with little or no additional cost. For example, Proposition 6.4 exemplifies how a classical 2-CFF built from a design can be enhanced by adding at most half as many tests to obtain much more information about the spread of a disease in a community using knowledge on how the individuals are partitioned into close-contact sub-communities.

## Conclusion and future work

In this work, we have explored group testing over hypergraphs, focusing on non-adaptive group testing using cover-free families (CFFs). We define vertex-identifying CFFs, edge-identifying CFFs, and intra-edge-screening CFFs, and explore their relationships in the construction of CFFs over hypergraphs. We consider the case of overlapping and non-overlapping edges and present constructions and upper bounds for each case. Finally, we define Swiss-army-knife CFFs, which are CFFs that hold several purposes. We show they can be naturally constructed via arrays, polynomials, and combinatorial designs.

The present paper and related work (De Bonis 2024; Gonen et al. 2022; Idalino 2019; Idalino and Moura 2022; Nikolopoulos et al. 2021b; Vorobyev 2022) are starting points to explore group testing over hypergraphs. The present paper and an early version (Idalino and Moura 2022) are, to the best of our knowledge, the first works to define and provide a general framework for studying cover-free families on hypergraphs; these have connections to problems in extremal set theory. In particular, the case of CFFs on graphs is the focus of a recent preprint by Parida and the second author (Parida and Moura 2025), where CFFs on graphs are proven to be “intermediate families” between Sperner families and 2-CFFs in terms of bounds. In Parida and Moura (2025), non-trivial asymptotic bounds for CFFs on specific families of graphs such as stars, paths and cycles are provided.

For future work, one could study more bounds and constructions for CFFs on uniform hypergraphs, which yield interesting combinatorial optimization problems with connections to extremal set theory. In addition, it would be useful to explore applications where CFFs on hypergraphs are effective, determining classes of hypergraphs that arise in these applications and exploring specific constructions for CFFs on these classes of hypergraphs.

## Data Availability

No datasets or code were generated or used during the current study. All findings are contained within the article.
